# Complications and mortality of typhoid fever: A global systematic review and meta-analysis

**DOI:** 10.1016/j.jinf.2020.10.030

**Published:** 2020-12

**Authors:** Christian S. Marchello, Megan Birkhold, John A. Crump

**Affiliations:** a Centre for International Health, University of Otago, PO Box 56, Dunedin 9016, New Zealand; b Department of Surgery, University of Maryland School of Medicine, Baltimore, MD 21201, USA

**Keywords:** Typhoid fever, Case fatality ratio, Meta-analysis, Mortality, Intestinal perforation

## Abstract

**Objectives:**

Updated estimates of the prevalence of complications and case fatality ratio (CFR) among typhoid fever patients are needed to understand disease burden.

**Methods:**

Articles published in PubMed and Web of Science from 1 January 1980 through 29 January 2020 were systematically reviewed for hospital or community-based non-surgical studies that used cultures of normally sterile sites, and hospital surgical studies of typhoid intestinal perforation (TIP) with intra- or post-operative findings suggestive of typhoid. Prevalence of 21 pre-selected recognized complications of typhoid fever, crude and median (interquartile range) CFR, and pooled CFR estimates using a random effects meta-analysis were calculated.

**Results:**

Of 113 study sites, 106 (93.8%) were located in Asia and Africa, and 84 (74.3%) were non-surgical. Among non-surgical studies, 70 (83.3%) were hospital-based. Of 10,355 confirmed typhoid patients, 2,719 (26.3%) had complications. The pooled CFR estimate among non-surgical patients was 0.9% for the Asia region and 5.4% for the Africa region. Delay in care was significantly correlated with increased CFR in Asia (*r* = 0.84; *p*<0.01). Among surgical studies, the median CFR of TIP was 15.5% (6.7–24.1%) per study.

**Conclusions:**

Our findings identify considerable typhoid-associated illness and death that could be averted with prevention measures, including typhoid conjugate vaccine introduction.

## Highlights

• Complications and death are considerable among hospitalized patients with typhoid fever.
• Case fatality ratio of typhoid fever was higher in Africa compared to Asia.
• Among studies in Africa, 20% of patients with typhoid intestinal perforation died.
• Delays in care were correlated with increased typhoid case fatality ratio in Asia.

## Introduction

Typhoid fever is caused by the organism *Salmonella enterica* subspecies *enterica* serovar Typhi (*Salmonella* Typhi); a systematic infection transmitted predominantly through water or food contaminated by human feces.^1^, ^2^, ^3^ Typhoid fever presents clinically across a spectrum of severity with a range of symptoms and signs including fever, abdominal pain, nausea, and vomiting, that make differentiating it from other febrile and gastrointestinal illnesses challenging.^2^ The ‘gold standard’ diagnostic method for typhoid fever is the culture of blood, bone marrow, or another normally sterile site. However, clinical microbiology services are not widely available in endemic areas and culture-based diagnosis has incomplete sensitivity.^4^ Additionally, delays in diagnosis and treatment occur as a result of barriers to care, such as difficulty accessing tertiary facilities because of delayed referral, distance, and the cost of healthcare.^5^, ^6^, ^7^

Timely and accurate diagnosis and treatment of typhoid fever in the community is needed to avert complications requiring hospitalization, and death.^2^ Typhoid complications include typhoid intestinal perforation (TIP), gastrointestinal hemorrhage, hepatitis, cholecystitis, myocarditis, shock, encephalopathy, pneumonia, and anemia.^1^^,^^2^ TIP and gastrointestinal hemorrhage are serious complications that are often fatal, even if managed surgically.^8^^,^^9^

Prevention of typhoid fever by improved sanitation and increased access to clean, safe water and food remains critical,^10^, ^11^, ^12^ but requires substantial investment over long time scales. Typhoid conjugate vaccine (TCV) has been pre-qualified and recommended by the World Health Organization for routine use^13^ and represents a tool to prevent typhoid illness and deaths in a short time horizon, complementing progress in sanitation, water, and food safety improvements.^14^ In typhoid-endemic countries, TCV pre-qualification allows for priority access and funding, removing important hurdles for vaccine introduction into routine immunization schedules.^15^

While previous systematic reviews have examined the case fatality ratio (CFR) of typhoid fever, they did not capture the substantial number of observational studies on typhoid fever published in recent years. Furthermore, some past reviews were restricted by location, population, or age.^10^^,^^16^, ^17^, ^18^, ^19^ In order to support country-level decisions on typhoid control, including TCV introduction, and to provide contemporary estimates of morbidity and mortality, we performed a systematic review and meta-analysis of the prevalence of complications and case fatality ratio (CFR) among patients with typhoid fever.

## Methods

### Search strategy

We systematically reviewed PubMed and Web of Science for published articles on the complications and mortality of typhoid fever. Each database was searched for key words of *Salmonella* Typhi, mortality, case fatality, died, death, complications, perforation, and hemorrhage (Supplementary Appendix A). Since a previous review reported on the mortality of typhoid fever prior to 1980,^20^ our search was limited to articles published from 1 January 1980 through 29 January 2020. We placed no restrictions on language, country, or demographics. We followed the Preferred Reporting Items for Systematic Reviews and Meta-Analyses (PRISMA; Supplementary Appendix B)^21^ and the protocol was registered with PROPSERO International Prospective Register of Systematic Reviews on 10 July 2020 (CRD42020166998). Ours was a study of published data and as such, institutional review board approval was not required.

### Study selection

We selected ‘non-surgical studies’ reporting the proportion of participants with *Salmonella* Typhi infection who had typhoid-associated complications or who died. In such studies, *Salmonella* Typhi infection was required to be ascertained by culture of a normally sterile site (e.g., blood). We also selected ‘surgical studies’ of only participants undergoing surgery for intestinal perforation. Surgical studies were included if gross intraoperative findings contained the keywords ‘terminal ileum,’ ‘antimesenteric perforation,’ or ‘confirmed at laparotomy’ to assign perforations as TIP.^8^^,^^9^ We also accepted postoperative criteria including the use of histopathology stains or immunohistochemistry to differentiate alternative causes for perforation (e.g., tuberculosis) and to attribute the cause of perforation to *Salmonella* Typhi. Consequently, we considered participants from non-surgical studies as having ‘confirmed,’ and those from surgical studies as having ‘probable,’ typhoid fever. Inclusion and exclusion criteria are summarized in Table 1.

**Table 1:** Inclusion and exclusion criteria for search strategy of complications and mortality of typhoid fever systematic review.

**Inclusion**	**Exclusion**
• Non-surgical studies using culture of normally sterile site specimens • Surgical studies of intestinal perforation using either: ○ Gross intraoperative findings with the keywords ‘terminal ileum’, ‘antimesenteric perforation’, or ‘confirmed at laparotomy’ ○ Postoperative findings using histopathology stains or immunohistochemistry to differentiate alternative causes for perforation • Article published after 1980 but data collected at any point • Hospital- or community-based study designs of active household or population-based surveillance, prospective observational, cross-sectional, case-control, retrospective medical record, or control arms of clinical trials	• Case reports, case series, policy reports, commentaries, editorials, and conference abstracts • Aggregate or summary data estimating case fatality ratio • Government passive surveillance reports • Used clinical indication (i.e., symptoms and signs), culture of a non-sterile site (e.g., stool or urine), or serology to classify a case of typhoid fever • Clear denominator was not available to calculate proportions

Titles and abstracts were downloaded from each database, imported into Endnote X8 (Clarivate Analytics, Boston, MA, USA), and combined into one reference list. Duplicates were removed by Endnote, and the de-duplicated list of articles was uploaded to the online systematic review tool Rayyan (Qatar Computing Research Institute, Doha, Qatar) for screening.^22^ Each subsequent process, including title and abstract review, full text review, and data abstraction, was performed in parallel by two authors (CSM and MB). A third author (JAC) was consulted when CSM and MB were unable to resolve discrepancies through discussion. The full text of 33 studies included in a systematic review on typhoid incidence were additionally screened.^23^ Data were then abstracted into a shared Google Sheets spreadsheet (Google LLC, Mountain View, CA, USA).

### Data abstraction

Abstracted study characteristics included the first author, publication year, article identifier (e.g., PubMed ID), year data collection started and ended, the city, district, or locality of the study, region and sub-region as classified by the United Nations (UN),^24^ type of normally sterile site cultured or study-specific TIP definition, whether participants were recruited from the community or a hospital, and if the study was non-surgical or surgical.

Data were abstracted for the mean and median fever, illness, or symptom duration prior to presentation; inclusion age and age range of participants; total number of confirmed or probable typhoid cases; number and type of complications; and number of deaths attributed to typhoid fever. When reported, we abstracted CFR data for MDR cases, defined by authors as infection with *Salmonella* Typhi resistant to chloramphenicol, ampicillin, and trimethoprim-sulfamethoxazole, and non-MDR cases, defined by authors as susceptible to at least one of the first-line antimicrobials. We also noted the proportion of male and female participants with TIP.

Data on duration of fever, duration of illness, and duration of symptoms prior to treatment were used as a proxy for delays in accessing care and subsequently combined as one metric of ‘delay in care.’ We categorized the ages of participants into three groups based on inclusion age and age range: ‘children’ were ≤15 years old, ‘adults’ >15 years, and ‘mixed ages’ were studies of both children and adult participants. Complications were classified two ways. First, we used a pre-selected list of 21 complications defined by Parry and colleagues.^1^ If a study mentioned any complication from the list, regardless of whether the study defined it as a typhoid complication, we abstracted the data. Second, we noted separately when a study specifically used the term ‘complication’ associated with typhoid fever. We did not abstract complications following surgery nor those attributed to the surgical procedure. If a study described an initial diagnosis, including pneumonia, hepatitis, and other syndromes that overlapped with complications of typhoid fever from our pre-selected list, it was not recorded as a complication due to lack of attribution to typhoid by the authors of the study. The final dataset was reviewed by a third author (JAC) for completeness and accuracy.

### Data analysis

For each non-surgical study, we divided the number of each pre-selected complication by the number of confirmed typhoid cases to calculate the prevalence of the specific complication. We divided the number of deaths attributed to typhoid fever by the total number of confirmed typhoid cases to calculate a CFR. We calculated the median and interquartile (IQR) range CFR for studies across each UN region and a pooled CFR estimate using a random effects model meta-analysis with MetaXL (Epigear International version 5.3). For pooled CFR estimates, we also stratified by UN sub-region and by age group. Among surgical studies of TIP, we calculated the CFR of TIP among probable typhoid cases and the prevalence of TIP among male and female participants.

Proportions were compared by Χ^2^ test, means by *t*-test, and the relationship between delay in care and CFR by Pearson's correlation coefficient (r), in R version 4.0.2 (R Foundation for Statistical Computing, Vienna, Austria) and considered significant if *p* <0.05. We assessed bias throughout the analyses by separating non-surgical and surgical analyses, by stratifying by region, sub-region, age, and study recruitment setting, and by heterogeneity using I^2^.

## Results

**Fig. 1:**
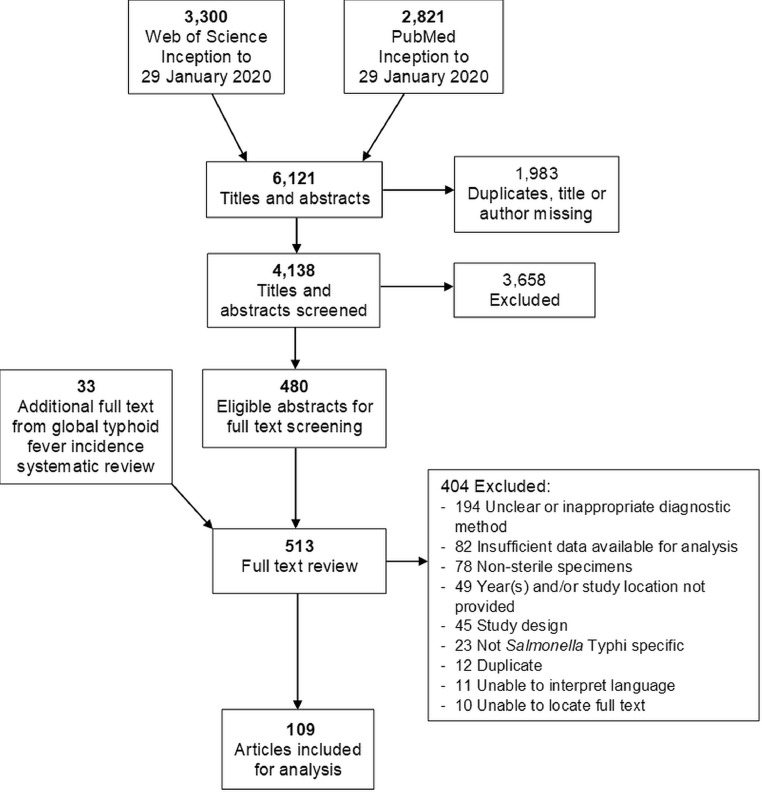
Preferred Reporting Items for Systematic Reviews and Meta-Analyses flow diagram of search strategy and selection of articles for mortality and complications of typhoid fever, 1965–2018.

Our search strategy returned 6,121 articles (Fig. 1). Of 513 full text articles reviewed, 404 were excluded. An unclear or inappropriate diagnostic method due to inadequate description of how a confirmed or probable typhoid case was defined was the most common reason for exclusion. We were unable to translate the language of 11 articles and we were unable to locate the full text of 10 articles. A total of 109 articles were included for analysis.^20^^,^^25^, ^26^, ^27^, ^28^, ^29^, ^30^, ^31^, ^32^, ^33^, ^34^, ^35^, ^36^, ^37^, ^38^, ^39^, ^40^, ^41^, ^42^, ^43^, ^44^, ^45^, ^46^, ^47^, ^48^, ^49^, ^50^, ^51^, ^52^, ^53^, ^54^, ^55^, ^56^, ^57^, ^58^, ^59^, ^60^, ^61^, ^62^, ^63^, ^64^, ^65^, ^66^, ^67^, ^68^, ^69^, ^70^, ^71^, ^72^, ^73^, ^74^, ^75^, ^76^, ^77^, ^78^, ^79^, ^80^, ^81^, ^82^, ^83^, ^84^, ^85^, ^86^, ^87^, ^88^, ^89^, ^90^, ^91^, ^92^, ^93^, ^94^, ^95^, ^96^, ^97^, ^98^, ^99^, ^100^, ^101^, ^102^, ^103^, ^104^, ^105^, ^106^, ^107^, ^108^, ^109^, ^110^, ^111^, ^112^, ^113^, ^114^, ^115^, ^116^, ^117^, ^118^, ^119^, ^120^, ^121^, ^122^, ^123^, ^124^, ^125^, ^126^, ^127^, ^128^, ^129^, ^130^, ^131^, ^132^

### Study characteristics

Among the 109 articles, one (0.9%) collected data in five countries,^90^ resulting in 113 study sites (Supplementary Appendix C). Among 113 study sites, data were collected from 1965 through 2018 and from every UN region; 62 (54.9%) in Asia, 44 (38.9%) in Africa, four (3.5%) in the Americas, two (1.8%) in Oceania, and one (0.9%) in Europe. Eighty-four (74.3%) sites recruited non-surgical typhoid fever participants and 29 (25.7%) were surgical studies of TIP. Among 84 non-surgical studies, 70 (83.3%) were hospital-based and 14 (16.7%) were community-based. There were 14,007 confirmed cases of typhoid fever with a median (IQR) of 64 (25–190) cases per study; 12,889 (92.0%) were from hospital-based and 1,118 (8.0%) from community-based studies. Among 29 surgical study sites, there were 2,926 probable cases of typhoid with a median of 58 (46–104) cases per study. Of the 16,933 total confirmed or probable cases, 11,973 (70.7%) were from Asia, 3,642 (21.5%) from Africa, 739 (4.4%) from Oceania, 554 (3.3%) from the Americas, and 25 (0.1%) from Europe. Sixty-seven (59.3%) of 113 study sites recruited participants of a mixed age, 35 (31.0%) recruited only children, and 11 (9.7%) only adults.

### Typhoid fever complications

Of the 84 non-surgical study sites, 56 (66.7%) reported at least one complication occurring from the list of pre-selected complications. Among 10,335 cases of confirmed typhoid fever, there were 2,719 (26.3%) complication events (Table 2). Delirium and anemia were the most prevalent complications, occurring in 705 (26.6%) of 2,648 and 1,017 (21.4%) of 4,756 confirmed cases, respectively. Eighty (1.3%) of 6,064 participants had TIP; 34 (0.7%) of 4,622 participants in Asia and 37 (7.6%) of 486 participants in Africa. Twenty-seven (32.1%) of 84 sites described unspecified complications for 669 (15.1%) of 4,442 confirmed cases. Asymptomatic electrocardiographic changes, impairment of coordination, pharyngitis, and chronic carriage were not reported from any of the included non-surgical study sites. Data on miscarriage were available in one study of pregnant women,^42^ occurring in one (16.7%) of six with confirmed typhoid fever. Although not in the Parry et al. list of typhoid complications, seizures or convulsions were reported among 108 (2.5%) of 4,349 typhoid patients.

**Table 2:** Complications of typhoid fever, by United Nations region, 1965–2018.

**Complications**^a^	**Africa**			**Americas**			**Asia**			**Oceania**			**Total**^b^		
	**n /**	**N**	**(%)**	**n /**	**N**	**(%)**	**n /**	**N**	**(%)**	**n /**	**N**	**(%)**	**n /**	**N**	**(%)**
**Abdominal**															
Intestinal perforation	37 /	486	(7.6)	4 /	217	(1.8)	34 /	4,622	(0.7)	5 /	739	(0.7)	80 /	6,064	(1.3)
Gastrointestinal hemorrhage	11 /	320	(3.4)	0 /	0	—	87 /	2,809	(3.1)	21 /	739	(2.8)	119 /	3,868	(3.1)
Hepatitis	10 /	157	(6.4)	1 /	9	(11.1)	104 /	2,389	(4.4)	17 /	739	(2.3)	132 /	3,294	(4.0)
Cholecystitis	1 /	55	(1.8)	0 /	0	—	10 /	913	(1.1)	0 /	365	(0.0)	11 /	1,333	(0.8)
**Cardiovascular**															
Asymptomatic electrocardiographic changes	ND			ND			ND			ND			ND		
Myocarditis	2 /	191	(1.0)	0 /	0	—	30 /	1,979	(1.5)	1 /	365	(0.3)	33 /	2,535	(1.3)
Shock	0 /	14	(0.0)	0 /	0	—	59 /	3,580	(1.6)	17 /	365	(4.7)	76 /	3,959	(1.9)
**Neuropsychiatric**															
Encephalopathy	0 /	0	—	0 /	0	—	98 /	2,460	(4.0)	4 /	365	(1.1)	102 /	2,825	(3.6)
Delirium	34 /	277	(12.3)	0 /	0	—	650 /	2,027	(32.1)	21 /	344	(5.8)	705 /	2,648	(26.6)
Psychotic states	2 /	50	(4.0)	2 /	217	(0.9)	28 /	1,438	(1.9)	0 /	0	—	32 /	1,705	(1.9)
Meningitis	6 /	347	(1.7)	1 /	9	(11.1)	13 /	1,625	(0.8)	0 /	0	—	20 /	1,981	(1.0)
Impairment of coordination	ND			ND			ND			ND			ND		
**Respiratory**															
Bronchitis	0 /	0	—	0 /	0	—	32 /	407	(7.9)	0 /	0	—	32 /	407	(7.9)
Pneumonia	4 /	191	(2.1)	7 /	226	(3.1)	43 /	1,416	(3.0)	18 /	374	(4.8)	72 /	2,207	(3.3)
**Hematologic**															
Anemia	132 /	311	(42.4)	52 /	226	(23.0)	683 /	3,516	(19.4)	150 /	703	(21.3)	1,017 /	4,756	(21.4)
Disseminated intravascular coagulation	0 /	0	—	0 /	0	—	98 /	660	(14.8)	1 /	374	(0.3)	99 /	1,034	(9.6)
**Other**															
Focal abscess	1 /	47	(2.1)	0 /	0	—	0 /	0	—	0 /	0	—	1 /	47	(2.1)
Pharyngitis	ND			ND			ND			ND			ND		
Miscarriage	0 /	0	—	0 /	0	—	1 /	6	(16.7)	0 /	0	—	1 /	6	(16.7)
Relapse	6 /	171	(3.5)	2 /	129	(1.6)	71 /	2,166	(3.2)	0 /	0	—	79 /	2,466	(3.2)
Chronic carriage	ND			ND			ND			ND			ND		
Seizure or convulsions^c^	14 /	125	(11.2)	0 /	0	—	94 /	4,224	(2.2)	0 /	0	—	108 /	4,349	(2.5)
**Total complications**	**260 /**	**689**	**(37.7)**	**69 /**	**226**	**(30.5)**	**2,135 /**	**8,681**	**(24.6)**	**255 /**	**739**	**(34.5)**	**2,719 /**	**10,335**	**(26.3)**
**Total Complications as described by study**	**116 /**	**348**	**(33.3)**	**24 /**	**327**	**(7.3)**	**401 /**	**3,028**	**(13.2)**	**128 /**	**739**	**(17.3)**	**669 /**	**4,442**	**(15.1)**

*a Complications from Parry et al. Table 1^1^ ND = No data. Data could not be abstracted as these complications were not described in any of the included articles.

**b Europe not shown due to the single study from Europe including participants diagnosed with stool and urine cultures, therefore it was not possible to distinguish complications among those diagnosed by culture of a normally sterile site. ^101^.

^cComplication not listed by Parry et al

### Outcomes of typhoid intestinal perforation

We identified 29 articles reporting surgical studies of TIP and an additional seven non-surgical studies provided data on CFR of TIP. Among the 36 combined surgical and non-surgical studies, 12 (33.3%) were in Asia, 23 (63.9%) in Africa, and one (2.8%) in the Americas. There were a total of 2,971 TIP cases, of which 2,921 (98.3%) were from surgical studies and 50 (1.7%) were from non-surgical studies. Of 2,971 TIP cases, 999 (33.6%) were in Asia, 1,967 (66.2%) in Africa, and 5 (0.2%) in the Americas. Of 2,971 TIP cases, 433 (14.6%) died. The median CFR of TIP across the 36 studies was 15.5% (6.7–24.1%).

Of 999 TIP cases in Asia, 46 (4.6%) died. The median CFR of TIP across 12 studies in Asia was 1.0% (0.0–8.4%). Of 1,967 TIP cases in Africa, 387 (19.7%) died. The median CFR of TIP across 23 studies in Africa was 20.0% (13.7–28.0%). Sex was available for 996 (99.7%) of 999 TIP cases in Asia; 704 (70.7%) were male compared to 292 (29.3%) female (Χ^2^=170.4; *p*<0.01). Sex was available for 1,826 (92.8%) of 1,967 TIP cases in Africa; 1,210 (66.3%) were male compared to 616 (33.7%) female (Χ^2^=193.2; *p*<0.01).

### Typhoid fever mortality

Seventy-nine (94.0%) of 84 non-surgical study sites reported on mortality. Among 13,303 confirmed typhoid cases from studies reporting mortality, 250 died, for a CFR of 1.9% (Table 3). The pooled CFR estimate (95% CI; heterogeneity I^2^) among 79 studies reporting on mortality of confirmed typhoid fever was 2.0% (1.4–2.8%; 83.9%). The pooled CFR estimates for the Asia, Africa, Oceania, the Americas, and Europe regions were 0.9% (0.6–1.3%; 63.4%), 5.4% (2.7–8.9%; 83.4%), 7.2% (0.0–20.4%; 97.2%), 6.7% (0.0–19.9%; 94.4%), and 1.0% (0.0–6.8%; incalculable), respectively. Data on outcomes for multi-drug resistant *Salmonella* Typhi infection, outcomes and sub-regions and sub-regional forest plots for South-eastern Asia, Southern Asia, Eastern Africa, and Western Africa, including stratification in these sub-regions by age groups, are provided in Supplementary Appendix D and E, respectively.

**Table 3:** Confirmed and probable cases of typhoid fever and case fatality ratio of confirmed typhoid fever, by United Nations region, 1965–2018.

	**Africa**	**Americas**	**Asia**	**Europe**	**Oceania**	**All regions**
**Confirmed and probable typhoid fever**						
Study locations	44	4	62	1	2	113
Confirmed and probable cases	3,642	554	11,973	30	739	16,938
Median (IQR) cases per study	50 (26–102.5)	113.5 (9.8–242.3)	79 (40–189)	a	369.5 (367.3–371.8)	63 (30–163)
						
**Mortality among confirmed cases**						
Study locations	22	3	51	1	2	84
Confirmed cases	1,700	544	10,295	25	739	13,303
Number of deaths	77	23	90	0	60	250
CFR confirmed typhoid fever	4.5	4.2	0.9	0.0	8.1	1.9
Median CFR (IQR)	4.2 (0.0–7.1)	9.2 (4.8–15.7)	0.0 (0.0–1.5)	0.0 (0.0)	8.1 (5.3–10.8)	0.2 (0.0–4.2)
Pooled CFR (95% CI)	5.4% (2.7–8.9%)	6.7% (0.0–19.9%)	0.9% (0.6–1.3%)	0.8% (0.0–5.7%)	7.2% (0.0–20.4%)	2.0% (1.4–2.8%)
						

*a No median; IQR = interquartile range; CI = confidence interval.

**Fig. 2:**
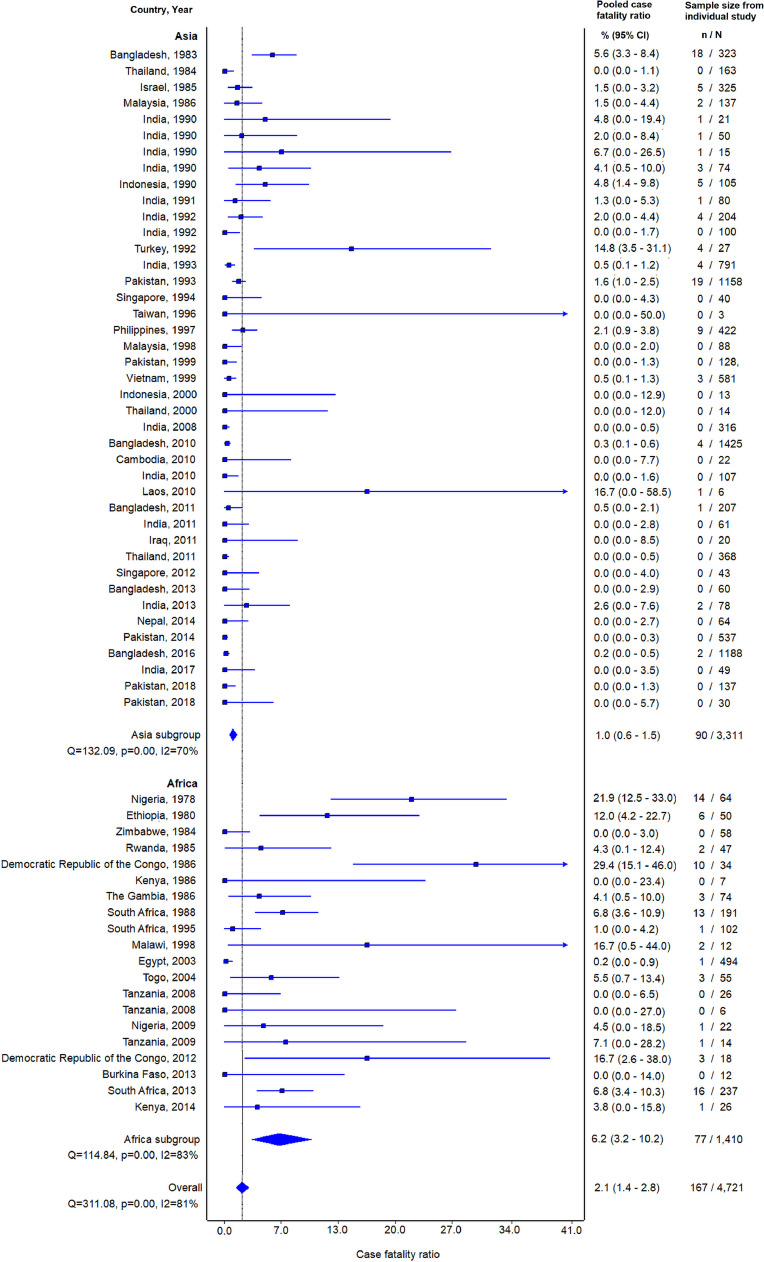
Forest plot of typhoid case fatality ratio among non-surgical hospital-based study sites in Asia and Africa, by year, 1978–2018.

Sixty-seven (84.8%) of 79 non-surgical study sites were hospital-based, and 12 (15.2%) were population or community-based studies.^37^^,^^38^^,^^60^^,^^73^^,^^84^^,^^90^^,^^96^^,^^118^ Ten (83.3%) of 12 non-hospital study sites were located in Asia and two (16.7%) in Africa; one each in Kenya^37^ and Burkina Faso.^60^ No deaths were reported among 866 confirmed typhoid cases in the 12 non-hospital sites compared to 250 (2.0%) of 12,437 hospital-based confirmed cases (Χ^2^=16.7; *p*<0.01). The pooled CFR estimate for non-surgical hospital-based study sites was 2.4% (1.6–3.3%; 85.9%) compared to 0.2% (0.0–0.7%; 0.0%) for non-hospital sites. The pooled CRF estimate among hospital-based sites in Asia was 1.0% (0.6–1.5%; 69.7%) compared to 6.2% (3.2–10.2%; 83.5%) among hospital-based sites in Africa (Fig. 2).

### Care delays and outcome

Of 84 non-surgical studies, 20 (23.8%) reported a mean or median duration of fever, illness, or symptoms prior to care, as well as CFR. Two studies stratified duration of fever, one by age^38^ and the other by sex,^66^ for a total of 22 estimates. Of 22 estimates, seven (31.8%) were from Africa,^30^^,^^37^^,^^59^^,^^66^^,^^81^^,^^122^ 12 (54.6%) from Asia,^36^^,^^38^^,^^41^^,^^72^^,^^84^^,^^96^^,^^98^^,^^102^^,^^105^^,^^109^^,^^131^ and one each (4.5%) were from the Americas,^97^ Europe,^101^ and Oceania.^108^ Among all 22 estimates, there was a statistically non-significant positive correlation of longer delay in care with increased CFR (*r* = 0.11; *p* = 0.64). Among the 12 estimates from Asia, there was a significant positive correlation between delay in care and CFR (*r* = 0.84; *p*<0.01) and a non-significant negative correlation between delay in care and CFR (−0.42; *p* = 0.35) among seven estimates from Africa. Scatterplots for delay in care are shown in Supplementary Appendix F.

Among 19 estimates in Asia and Africa, the mean (range) delay in care was 7.5 (2.0–16.4) days in Asia and 9.4 (6.7–11.0) days in Africa (*p* = 0.19). Among the 17 hospital-based estimates reporting delay in care metrics, the mean (range) delay was 9.3 (5.0–16.4) days, compared with 5.3 (2.0–10.4) days among five community-based estimates (*p* = 0.03).

## Discussion

Our systematic review of published literature from 1980 through 2020 of predominantly hospitalized typhoid fever patients demonstrates a substantial prevalence of typhoid complications and death. We estimated a CFR of 2.0%, with significant variation by UN region. A considerable proportion of hospitalized patients with typhoid experienced complications. At the same time, delays in care, as measured by duration of fever or illness before presentation, were associated with increased CFR. However, we also identified significant differences in prevalence of complications and CFR between community-based and hospital-based studies, suggesting a bias towards poorer outcomes in hospital-based studies.

Among hospital-based non-surgical studies, the CFR of typhoid fever was significantly higher in Africa compared to Asia. Although there was no association between delay in care and CFR in Africa, our ability to detect an association was influenced by fewer data from Africa compared with Asia, where a statistically significant positive correlation was identified. Despite Africa comprising 39% of included study sites, it accounted for only 12% of all confirmed typhoid cases in our review. The limited data available on confirmed typhoid fever cases and smaller sample sizes of African studies may be due to lower typhoid incidence,^23^ compounded by the lack of capacity of many health care centers in Africa to obtain and perform blood cultures.^133^, ^134^, ^135^ Delays were longer in hospital-based sites compared to community-based sites, and such delays in diagnosis, appropriate treatment, and management of complications may contribute to the higher CFR identified among hospital studies. Others have linked duration of illness prior to hospitalization with increased prevalence of typhoid complications.^19^

Intestinal perforation was a common complication and an important contributor to typhoid mortality, especially in African studies where one in five patients with TIP died. A lack of access to surgical services and resources for post-operative management and intensive care, if needed, likely contribute to the high CFR seen among TIP patients.^48^^,^^123^^,^^133^^,^^136^ The prevalence of TIP as a complication of typhoid fever in non-surgical studies may be underestimated by our review, as some such studies were often done solely on medical wards or in hospitals that lacked surgical facilities.

A substantial limitation of our review was the preponderance of hospital-based studies. This introduced a bias towards higher prevalence of complications and higher CFR. However, care seeking behavior for febrile illnesses is not driven exclusively by severity. In some settings, poor transportation infrastructure, cost of healthcare, and difficulty in obtaining referrals can result in the sickest patients not reaching care.^5^, ^6^, ^7^ Alternatively, community-based studies alter the outcome towards the null by enhancing typhoid diagnosis and management, allowing early treatment before progression to severe and complicated disease.^137^ We attempted to limit other selection biases in non-surgical studies by stratifying by setting type, region and sub-region, and age, and by including only those that used culture of normally sterile sites to confirm typhoid fever. Among surgical studies, misclassification of non-typhoid causes of intestinal perforation as TIP are increasingly recognized.^138^ We were limited by the use of intra- and postoperative findings in classifying ileal perforations as TIP. We attempted to address this limitation by abstracting and presenting the criteria defining a case of TIP by study. Heterogeneity was high for pooled estimates of all studies. This was anticipated given the range of years, study designs, location, and age groups of included studies. However, when stratified by sub-region and by age group, heterogeneity was much lower.

Although our understanding of typhoid fever morbidity and mortality could be improved with more robust, community-based active surveillance studies, we demonstrate considerable typhoid fever morbidity and mortality that could be averted with prevention efforts. TCV represents a means to make rapid gains in prevention of typhoid fever complications and death.

## Author contributions

JAC conceived the study. CSM and JAC developed the research protocol. CSM submitted the review to PROSPERO and performed the literature search. CSM and MB screened titles and abstracts, reviewed full texts, and performed data abstraction. JAC resolved discrepancies and reviewed the final dataset. CSM performed data analyses and prepared the first manuscript draft. MB prepared the abstract, ‘Research in Context’, and gave feedback on the first and subsequent drafts. JAC provided major revisions and comments to the first draft. All authors contributed to final edits and revisions prior to submission.

## Declaration of Competing Interest

None.

## Funding

This work was supported by Bill & Melinda Gates Foundation (BMGF) [grant OPP1151153], to JAC and CSM. JAC also received support from BMGF [grant numbers OPP1125993 and OPP1158210], the US National Institutes of Health [grant number R01AI121378], and the New Zealand Health Research Council through the e-ASIA Joint Research Program [grant number 16/697]. MB received support from the US National Institutes of Health [grant number T32 DK067872].

## Role of the funder

The funders of the study had no role in study design, data collection, data analysis, data interpretation, or writing of the report. The corresponding author had full access to all the data in the study and had final responsibility for the decision to submit for publication.

## Appendix Supplementary materials

Image, application 1
