# Supplementary material for: Complications and mortality of typhoid fever: A global systematic review and meta-analysis
Source: J Infect. 2020 Dec;81(6):902–10. doi: 10.1016/j.jinf.2020.10.030 (PMC7754788; doi:10.1016/j.jinf.2020.10.030)
Supplement: Supplementary file 1 [file mmc1.docx]

**Supplementary Appendix**

Supplement to: Complications and mortality of typhoid fever: a global systematic review and meta-analysis

**Appendix A: Search strategy**

**PubMed Search 29 January 2020**

(Typhi OR typhoid) AND (mortality OR "morbidity"[All Fields] OR "morbidity"[MeSH Terms] OR died or fatal* or complicat* or perforat* or bleeding or hemorr* or haemorr*) AND ("1980/01/01"[PDAT] : "3000/12/31"[PDAT]) AND hasabstract[text]

**Results: 2,821**

**Web of Science 29 January 2020**

**
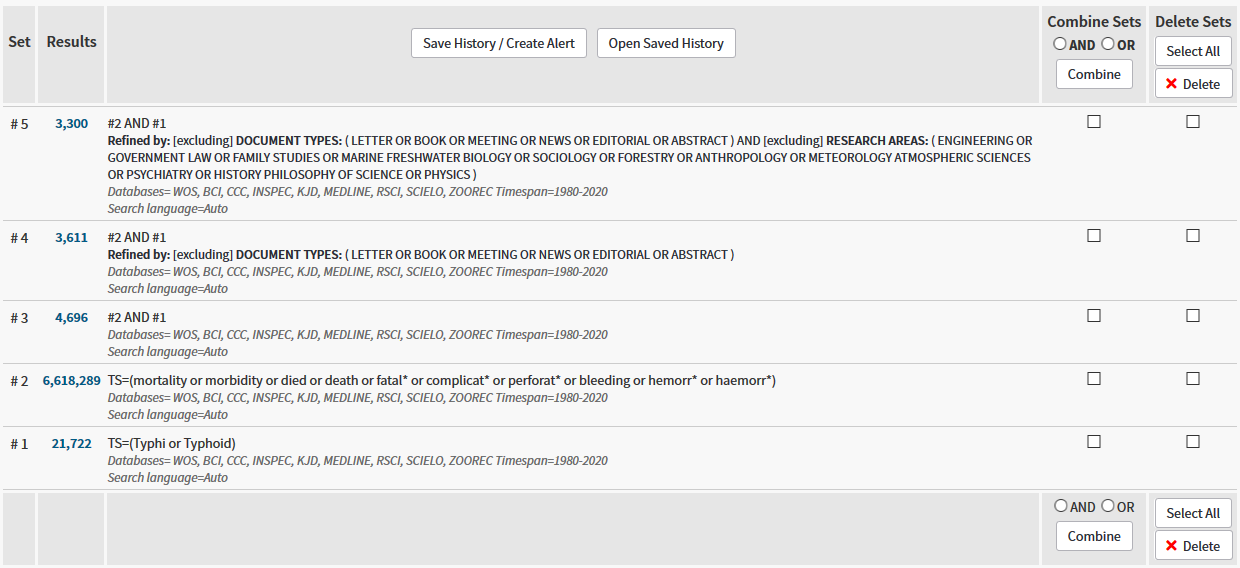
**

**Results: 3,300**

**Appendix B: Preferred Reporting Items for Systematic Reviews and Meta-Analyses (PRISMA) checklist**

| **Section/topic** | **#** | **Checklist item** | **Reported on page #** |
| --- | --- | --- | --- |
| **TITLE** | | |  |
| Title | 1 | Identify the report as a systematic review, meta-analysis, or both. | 1 |
| **ABSTRACT** | | |  |
| Structured summary | 2 | Provide a structured summary including, as applicable: background; objectives; data sources; study eligibility criteria, participants, and interventions; study appraisal and synthesis methods; results; limitations; conclusions and implications of key findings; systematic review registration number. | 3-4 |
| **INTRODUCTION** | | |  |
| Rationale | 3 | Describe the rationale for the review in the context of what is already known. | 5-6 |
| Objectives | 4 | Provide an explicit statement of questions being addressed with reference to participants, interventions, comparisons, outcomes, and study design (PICOS). | 5-6 |
| **METHODS** | | |  |
| Protocol and registration | 5 | Indicate if a review protocol exists, if and where it can be accessed (e.g., Web address), and, if available, provide registration information including registration number. | 6 |
| Eligibility criteria | 6 | Specify study characteristics (e.g., PICOS, length of follow-up) and report characteristics (e.g., years considered, language, publication status) used as criteria for eligibility, giving rationale. | 6-7 |
| Information sources | 7 | Describe all information sources (e.g., databases with dates of coverage, contact with study authors to identify additional studies) in the search and date last searched. | 6-7 |
| Search | 8 | Present full electronic search strategy for at least one database, including any limits used, such that it could be repeated. | Appendix A |
| Study selection | 9 | State the process for selecting studies (i.e., screening, eligibility, included in systematic review, and, if applicable, included in the meta-analysis). | 7 |
| Data collection process | 10 | Describe method of data extraction from reports (e.g., piloted forms, independently, in duplicate) and any processes for obtaining and confirming data from investigators. | 7-8 |
| Data items | 11 | List and define all variables for which data were sought (e.g., PICOS, funding sources) and any assumptions and simplifications made. | 7-8 |
| Risk of bias in individual studies | 12 | Describe methods used for assessing risk of bias of individual studies (including specification of whether this was done at the study or outcome level), and how this information is to be used in any data synthesis. | 9 |
| Summary measures | 13 | State the principal summary measures (e.g., risk ratio, difference in means). | 9 |
| Synthesis of results | 14 | Describe the methods of handling data and combining results of studies, if done, including measures of consistency (e.g., I^2^) for each meta-analysis. | 9 |

**Appendix C: Study characteristics of 109 included articles**

**Table S1. Characteristics of 109 included studies of global mortality and complications of typhoid fever by United Nations classification of geographic region and sub-region, 1965-2018**

| **UN Region and sub-region** | **Country [reference]** | **City, District, or Locality** | **Year(s) of data collection** | **Age group** | **Criteria for defining a case of typhoid fever** | **Population** |
| --- | --- | --- | --- | --- | --- | --- |
| **Africa** |  |  |  |  |  |  |
| Eastern Africa | Ethiopia^1^ | Addis Ababa | 1975-1980 | Mixed | Blood culture | NSH |
|  | Kenya^2^ | Nairobi | 1986 | Children | Blood culture | NSH |
|  | Kenya^3^ | Kilifi | 1998-2014 | Mixed | Blood culture | NSH |
|  | Kenya^4^ | Kibera | 2006-2009 | Mixed | Blood culture | NSC |
|  | Malawi^5^ | Blantyre | 1997-1998 | Children | Blood culture | NSH |
|  | Rwanda^6^ | Kigali | 1984-1985 | Children | Blood culture | NSH |
|  | Rwanda^7^ | Kigali | 2014-2015 | Mixed | Intraoperative findings of antimesenteric perforation of the ileum | S |
|  | Tanzania^8^ | Mwanza | 2006-2011 | Mixed | Intraoperative findings of oval perforation on the antimesenteric border of the intestine and an acutely inflamed and edematous intestine | S |
|  | Tanzania^9^ | Moshi | 2007-2008 | Mixed | Blood culture | NSH |
|  | Tanzania^10^ | Moshi | 2007-2008 | Children | Blood culture | NSH |
|  | Tanzania^11^ | Muheza | 2008-2009 | Children | Blood culture | NSH |
|  | Uganda^12^ | Karamoja | 2010-2016 | Mixed | Presence of typical ulcers on the antimesenteric side of the terminal ileum | S |
|  | Zimbabwe^13^ | Harare | 1984-1984 | Children | Blood culture | NSH |
| Middle Africa | Democratic Republic of the Congo^14^ | Kimpese | 1982-1986 | Mixed | Blood, CSF, and synovial fluid culture | NSH |
|  | Democratic Republic of the Congo^15^ | Bwamanda | 2011-2012 | Mixed | Blood culture | NSH |
| Northern Africa | Egypt^16^ | Upper Egypt, Delta region, Coastal region, Cairo Metropolitan | 1999-2003 | Mixed | Blood culture | NSH |
| Southern Africa | South Africa^17^ | Durban | 1979-1983 | Children | Biopsy specimens obtained at laparotomy for histological confirmation and perforation of the ileum at autopsy | S |
|  | South Africa^18^ | Durban | 1987-1988 | Mixed | Blood culture and histology | NSH |
|  | South Africa^19^ | Durban | 1993-1995 | Mixed | Blood culture | NSH |
|  | South Africa^20^ | National | 2003-2013 | Mixed | Blood culture | NSH |
| Western Africa | Burkina Faso^21^ | Tenkodogo | 2010-2014 | Mixed | Intraoperatively with location of the perforation on the antimesenteric edge of the terminal ileum | S |
|  | Burkina Faso^22^ | Nanoro | 2012-2013 | Children | Blood culture | NSH |
|  | Burkina Faso^23^ | Nanoro | 2013-2014 | Children | Blood culture | NSC |
|  | Ghana^24^ | Berekum | 1978-1989 | Mixed | Typical operative findings of antimesenteric perforation of the distal ileum | S |
|  | Ghana^25^ | Asante-Akyim | 1982-1987 | Mixed | Operative finding of typical perforated typhoid ulcer at the antimesenteric border of the terminal ileum | S |
|  | Ghana^26^ | Berekum | 1990-1992 | Mixed | Operative findings of antimesenteric perforation of the distal ileum | S |
|  | Ghana^27^ | Kumasi | 1995-1996 | Children | Intraoperatively by typical findings of antimesenteric perforations of the ileum | S |
|  | Ghana^28^ | Kumasi | 2002-2005 | Adults | Operative findings of ileal perforations on the antimesenteric border | S |
|  | Ghana^29^ | Accra | 2009-2012 | Mixed | Findings of perforation at the antimesenteric border of the terminal ileum during surgery or post-mortem examinations | S |
|  | Nigeria^30^ | Ibadan | 1976-1978 | Children | Blood culture | NSH |
|  | Nigeria^31^ | Ibadan | 1989-1990 | Mixed | Operative findings of ileal perforation, and an acutely inflamed and oedematous terminal ileum with associated peritoneal soilage | S |
|  | Nigeria^32^ | Sagamu | 1990-2004 | Adults | Operative findings of oval perforation on the antimesenteric border of the jejunum or/and ileum | S |
|  | Nigeria^33^ | Benin City | 1993-2007 | Children | Confirmed at operation with perforations located at the terminal ileum | S |
|  | Nigeria^34^ | Jos | 1994-2003 | Mixed | Operative findings of longitudinal perforations in the jejunum and ileum as well as between the taenia coli in the colon | S |
|  | Nigeria^35^ | Jos | 1996-2005 | Children | Intraoperative diagnosis of typical perforated ulcers on the anti-mesenteric border on the intestine (oval in shape, with long axes disposed along the longitudinal axis of the bowel) | S |
|  | Nigeria^36^ | Kano | 1997-2003 | Mixed | Histology of excised ulcers or the finding of typical ulcers on the anti-mesenteric border with aggregation of lymphoid patches | S |
|  | Nigeria^37^ | Enugu | 1999-2007 | Mixed | Intraoperative finding of the classic antimesenteric ileal perforation with absence of omental reaction | S |
|  | Nigeria^38^ | Azare | 2004-2008 | Children | Intraoperative diagnosis of perforation, antimesenteric location | S |
|  | Nigeria^39^ | Ile-Ife | 2005-2013 | Children | Intraoperative finding of an acutely inflamed ileum (often terminal ileum) with perforation at the antimesenteric border | S |
|  | Nigeria^40^ | Calabar | 2006-2015 | Children | Intraoperatively by surgical finding of anterior mesenteric perforation of the distal ileum | S |
|  | Nigeria^41^ | Enugu | 2007-2009 | Mixed | Confirmed by operative findings, all were typically laid along the antimesenteric border of the ileum. Histopathological examination of the edge of the ileal perforation specimen was also a form of retrospective diagnostic confirmation | S |
|  | Nigeria^42^ | Abuja; Nyanya District; Keffi | 2008-2009 | Children | Blood culture | NSH |
|  | The Gambia^43^ | Fajara | 1981-1986 | Mixed | Blood culture | NSH |
|  | Togo^44^ | Lome | 1995-2004 | Children | Blood culture | NSH |
| **Americas** |  |  |  |  |  |  |
| Caribbean | Haiti^45^ | Deschapelles | 1988-1991 | Mixed | Blood culture | NSH |
| Central America | Mexico^46^ | Mexico City | 1983-1986 | Mixed | Terminal ileum perforations, ulcerated typhoid ileitis, or acute abdomen during the course of severe, toxic, typhoid fever | S |
| South America | Chile^47^ | Not provided | 1982-1988 | Mixed | Blood culture | NSH |
|  | Peru^48^ | Trujillo | 1965-1979 | Mixed | Blood culture | NSH |
| **Asia** |  |  |  |  |  |  |
| Eastern Asia | Taiwan^49^ | Kaohsiung City | 1991-1996 | Adults | Blood culture | NSH |
|  | China; India; Indonesia; Pakistan; Vietnam^50^ | Hechi; Kolkata; North Jakarta; Karachi; Hue | 2001; 2003; 2002; 2002; 2002-2002; 2004; 2003; 2004; 2003 | Mixed | Blood culture | NSC |
| Southern-eastern Asia | Cambodia^51^ | Siem Reap province | 2009-2010 | Children | Blood culture | NSH |
|  | Indonesia^52^ | Semarang | 1989-1990 | Mixed | Blood and bone marrow culture | NSH |
|  | Indonesia^53^ | Jayapura | 1997-2000 | Mixed | Blood culture | NSH |
|  | Laos^54^ | Vientiane | 2006-2010 | Pregnant women | Blood culture | NSH |
|  | Malaysia^55^ | Kelantan | 1984-1986 | Children | Blood culture | NSH |
|  | Malaysia^56^ | Kelantan | 1993-1998 | Children | Blood culture | NSH |
|  | Philippines^57^ | Tagbilaran City | 1994-1997 | Mixed | Blood culture | NSH |
|  | Singapore^58^ | Singapore | 1990-1994 | Children | Blood culture | NSH |
|  | Singapore^59^ | Singapore | 2006-2012 | Mixed | Blood culture | NSH |
|  | Thailand^60^ | Bangkok | 1977-1984 | Children | Blood culture | NSH |
|  | Thailand^61^ | Bangkok | 1986-2000 | Children | Blood culture | NSH |
|  | Thailand^62^ | Songkhla province | 2009-2011 | Children | Blood culture | NSH |
|  | Vietnam^63^ | Dong Thap Province | 1995-1996 | Mixed | Blood culture | NSC |
|  | Vietnam^64^ | Ho Chi Minh City; Cao Lanh | 1993;1997-1995;1999 | children | Blood and bone marrow cultures | NSH |
| Southern Asia | Bangladesh^65^ | Dhaka | 1975-1983 | Mixed | Blood culture | NSH |
|  | Bangladesh^66^ | Dhaka | 2000-2001 | Mixed | Blood culture | NSC |
|  | Bangladesh^67^ | Dhaka | 2003-2004 | Mixed | Blood culture | NSC |
|  | Bangladesh^68^ | Dhaka | 2004-2016 | Mixed | Blood culture | NSH |
|  | Bangladesh^69^ | Dhaka | 2009-2011 | Mixed | Blood culture | NSH |
|  | Bangladesh^70^ | Dhaka | 2009-2013 | Mixed | Blood culture | NSH |
|  | Bangladesh^71^ | Dhaka | 2010 | Mixed | Blood culture | NSH |
|  | India^72^ | Kashmir | 1988-1993 | Adults | Blood culture | NSH |
|  | India^73^ | West Bengal | 1989-1990 | Mixed | Blood culture | NSH |
|  | India^74^ | Pondicherry | 1990 | Children | Blood culture | NSH |
|  | India^75^ | New Delhi | 1990 | Children | Blood culture | NSH |
|  | India^76^ | Manipal | 1990-1991 | Mixed | Blood culture | NSH |
|  | India^77^ | Calcutta | 1990-1992 | Children | Blood culture | NSH |
|  | India^78^ | Bombay | 1990 | Mixed | Blood culture | NSH |
|  | India^79^ | Bombay | 1990-1992 | Children | Blood culture | NSH |
|  | India^80^ | New Delhi | 1995-1996 | Mixed | Blood culture | NSC |
|  | India^81^ | New Delhi | 2001-2013 | Mixed | Blood culture | S |
|  | India^82^ | New Delhi | 2001-2003 | Mixed | Blood culture | NSH |
|  | India^83^ | Kolkata | 2004 | Mixed | Blood culture | NSC |
|  | India^84^ | North India | 2005-2010 | Mixed | Blood culture | NSH |
|  | India^85^ | Chennai | 2005-2008 | Children | Blood culture | NSH |
|  | India^86^ | Kerala | 2011-2013 | Mixed | Blood culture | NSH |
|  | India^87^ | Ahmedabad | 2011 | Mixed | Blood culture | NSH |
|  | India^88^ | Chandigarh, Gurugram, Vellore, Kolkata, and Manipal | 2014-2015 | Mixed | Blood culture | NSH |
|  | India^89^ | Puducherry | 2016-2017 | Mixed | Blood culture | NSH |
|  | Nepal^90^ | Kathmandu | 1981-1988 | Mixed | Confirmed at laparotomy with antimesenteric perforations of the terminal ileum | S |
|  | Nepal^91^ | Kathmandu | 2002-2003 | Adults | Blood culture | NSH |
|  | Nepal^92^ | Dhulikhel | 2012-2014 | Mixed | Blood culture | NSH |
|  | Pakistan^93^ | Karachi | 1988-1993 | Children | Blood culture | NSH |
|  | Pakistan^94^ | Karachi | 1989-1999 | Adult females | Blood culture | NSH |
|  | Pakistan^95^ | Karachi | 2002-2004 | Mixed | Blood culture | NSC |
|  | Pakistan^96^ | Karachi | 2007-2008 | Children | Blood culture | NSC |
|  | Pakistan^97^ | Karachi and Hyderabad | 2012-2014 | Mixed | Blood culture | NSH |
|  | Pakistan^98^ | Islamabad | 2015-2018 | Mixed | Blood culture | NSH |
|  | Pakistan^99^ | Karachi | 2016-2018 | Mixed | Operative finding, histopathological examination of the edges of perforation | S |
|  | Pakistan^100^ | Karachi | 2018 | Children | Blood culture | NSH |
| Western Asia | Iraq^101^ | Sulaimania | 2011 | Mixed | Blood culture | NSH |
|  | Israel^102^ | Be'er Sheva and Afula | 1984-1985 | Mixed | Blood culture | NSH |
|  | Turkey^103^ | Erzurum | 1978-2004 | Mixed | Intraoperatively by the typical findings of antimesenteric perforations of the ileum, and postoperatively by the pathologic examination that showed histological evidence of typhoid inflammation in the tissue | S |
|  | Turkey^104^ | Ankara | 1982-1992 | Children | Blood culture | NSH |
|  | Turkey^105^ | Diyarbakir | 1994-2005 | Adults | Distal ileum and antimesenteric perforation, confirmed histopathologically | S |
|  | Turkey^106^ | Diyarbakir | 1994-1998 | Adults | Blood culture or intraoperative findings of anterior mesenteric perforation of the distal ileum | S |
| **Europe** |  |  |  |  |  |  |
| Southern Europe | Spain^107^ | Pontevedra | 1990-1992 | Mixed | Blood culture | NSH |
| **Oceania** |  |  |  |  |  |  |
| Melanesia | Fiji^108^ | National | 2014-2015 | Mixed | Blood culture | NSH |
|  | Papua New Guinea^109^ | Goroka | 1986-1989 | Adults | Blood and bone marrow culture | NSH |

UN = United Nations; NSH = Non-surgical hospital-based study; NSC = Non-surgical community-based study; S = Surgical study

**Appendix D: Multi-drug resistance and outcome**

Thirty-nine (46.4%) of 84 non-surgical studies provided some data on antimicrobial resistance. Two studies provided mortality data for both MDR and non-MDR cases using the standard definition for MDR.^66,67^ Both studies were conducted in Bangladesh and no deaths were recorded among the combined 43 MDR cases or the 36 non-MDR cases. One study did not find a statistically significant difference (p=0.34) in mortality between MDR and drug susceptible cases, but did not define which antimicrobials comprised each group.^93^ In one study from India in 1990, there were no deaths among 17 patients with MDR typhoid infection, defined as resistance to two or more drugs, and one death in the chloramphenicol sensitive group.^74^ Another study from India in 1990 reported one death in the MDR group, defined as resistance to ‘conventional drugs’, and no deaths in the susceptible group, with no description for which antimicrobials were susceptible.^75^ Neither study from India provided a statistical comparison.

**Appendix E: CFR sub-regional analysis and Figures S1-S4**

Among sub-regions in Asia, the pooled CFR estimate in the South-eastern Asia sub-region was 0.8% (0.3-1.6%; 49.2%) (Figure S1). The pooled CFR estimate among children in South-eastern Asia was 0.3% (0.0-0.7%; 0.0%) and 1.3% (0.3-2.7%; 54.9%) among adults and mixed ages. The pooled CFR estimate in Southern Asia was 0.8% (0.4-1.2%; 68.1%) (Figure S2). The pooled CFR estimate among children in Southern Asia was 0.7% (0.1-1.7%; 63.2%) and 0.7% (0.3-1.3%; 68.4%) among adults and mixed ages.

Among sub-regions in Africa, the pooled CFR estimate in the Eastern Africa sub-region was 3.6% (0.7-8.1%; 64.9%) (Figure S3). The pooled CFR estimate among children in Eastern Africa was 2.4% (0.2-6.3%; 10.8%) and 4.9% (0.0-13.2%; 81.1%) among adults and mixed ages. In Western Africa, the pooled CFR estimate was 6.3% (1.5-13.6%; 67.7%) (Figure S4). Among children in Western Africa, the pooled CFR was 6.4% (0.7-16.1%; 69.7%) and 4.1% (0.5-10.0%; I^2^ uncalculable) among adults and mixed ages. Three studies in Southern Africa reported 30 (5.7%) of 530 confirmed cases died and had a pooled CFR estimate of 4.7% (1.7-8.9%; 72.4%).^18–20^ In two studies from Middle Africa in the Democratic Republic of the Congo, 13 (25.0%) of 52 confirmed cases died.^14,15^

**Figure S1. Forest plot of the case fatality ratio of typhoid fever in the South-eastern Asia sub-region by age group, 1984-2012**

**
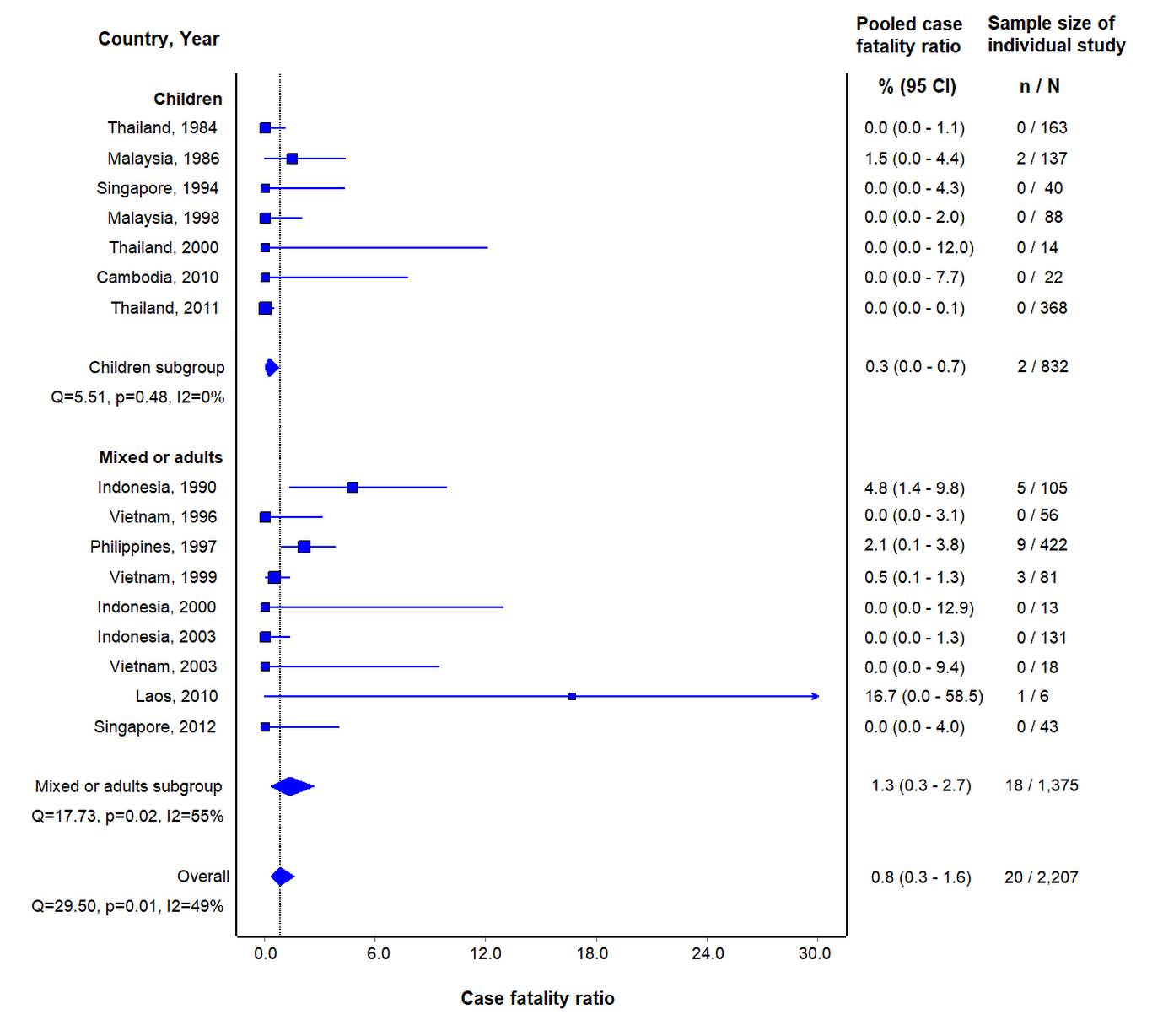
**

**Figure S2. Forest plot of the case fatality ratio of typhoid fever in the Southern Asia sub-region by age group, 1990-2018**

**
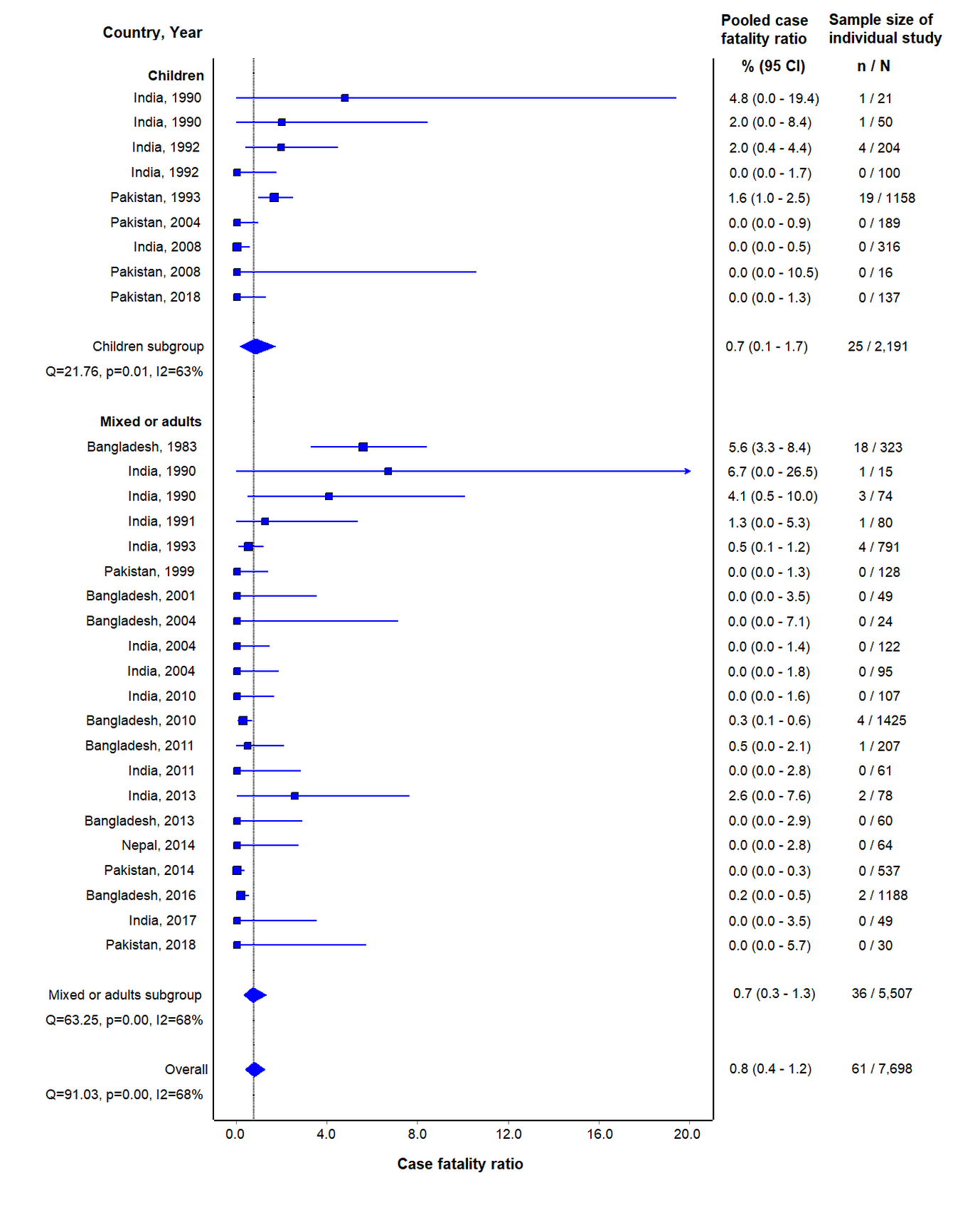
**

**Figure S3. Forest plot of the case fatality ratio of typhoid fever in the Eastern Africa sub-region by age group, 1980-2014**

**
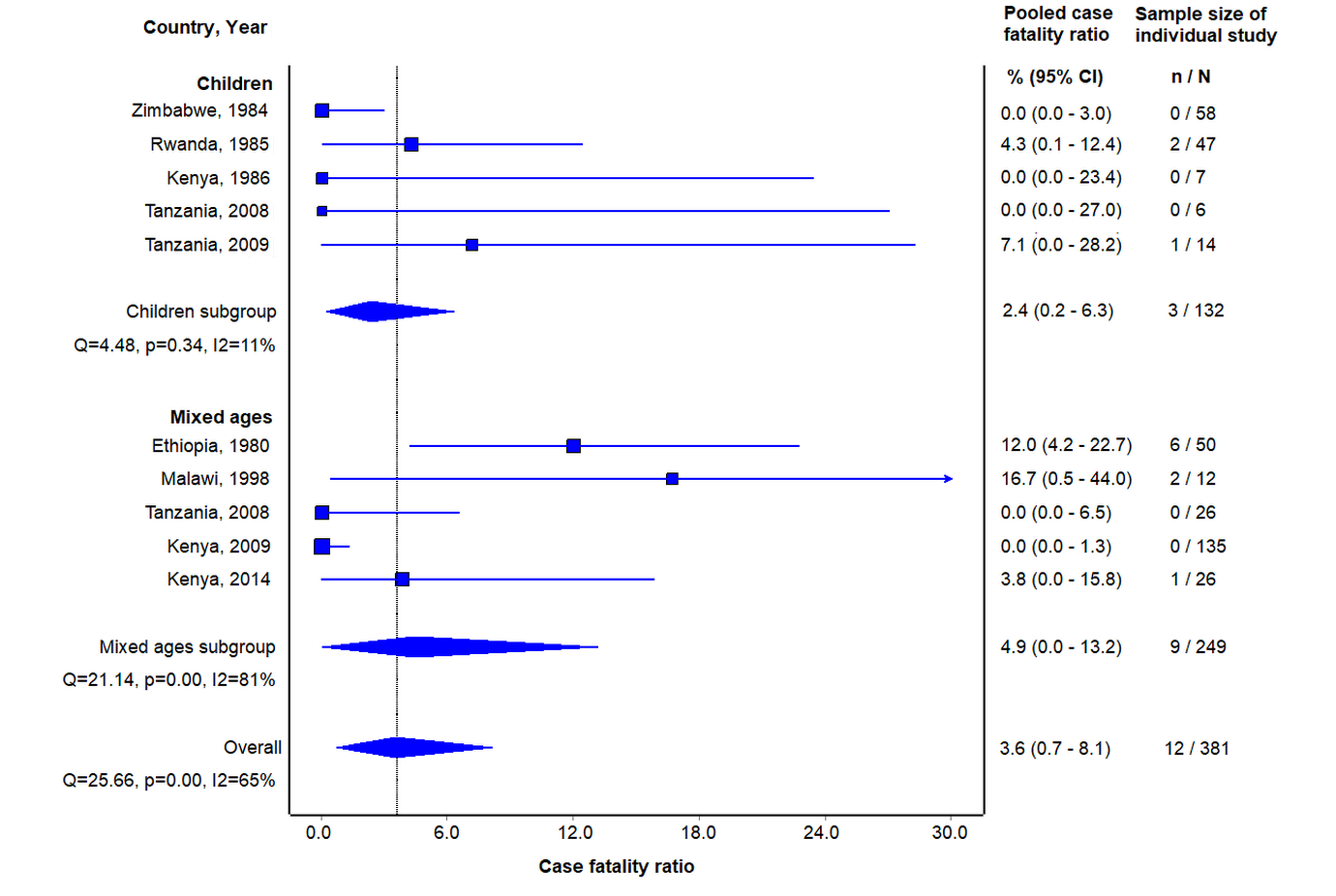
**

**Figure S4. Forest plot of the case fatality ratio of typhoid fever in the Western Africa sub-region by age group, 1978-2014**

**
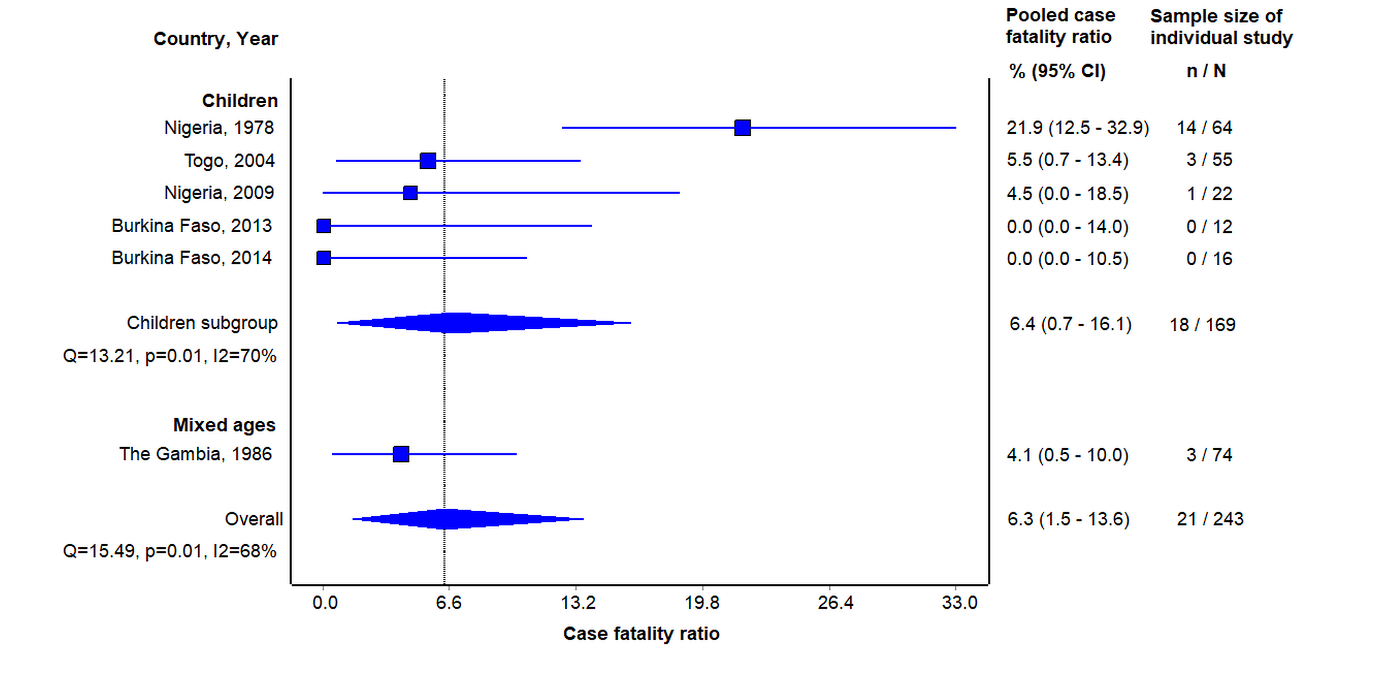
**

**Appendix F: Scatterplots of case fatality ratio against delay in care**

**Figure S5. Scatterplot of case fatality ratio against delay in care with trendline, 22 eligible estimates from all UN regions**

**
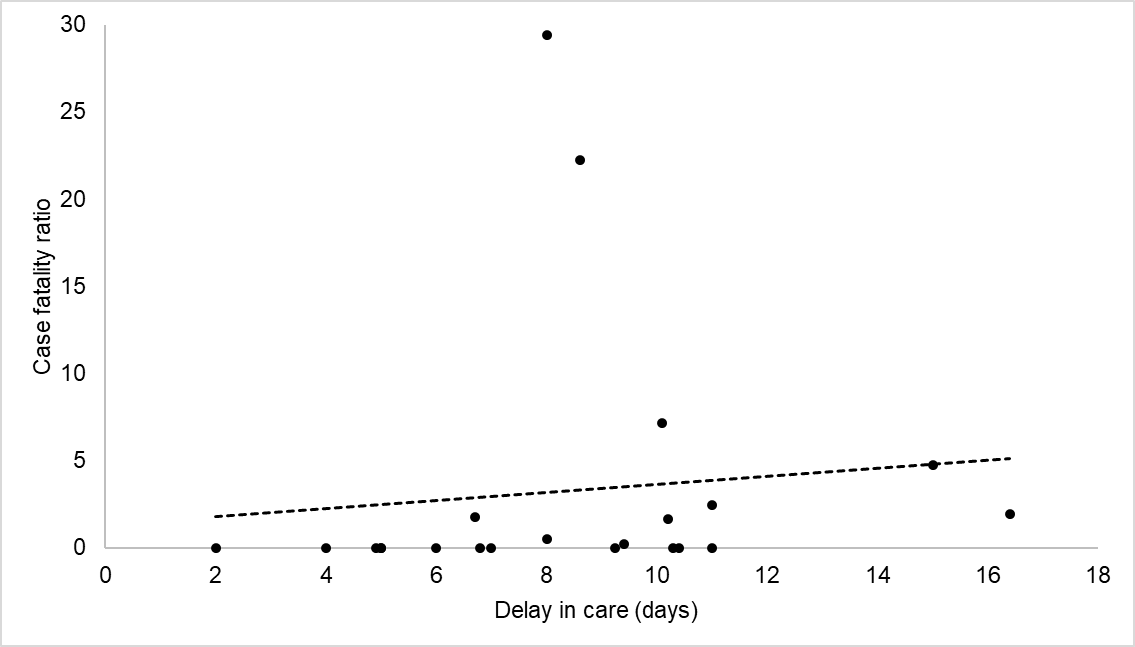
**

Trendline: y = 0.2337x + 1.3117

Pearson’s r = 0.1052 (p=0.6412)

**Figure S6. Scatterplot of case fatality ratio against delay in care with trendline, 19 eligible estimates from Asia and Africa regions**

**
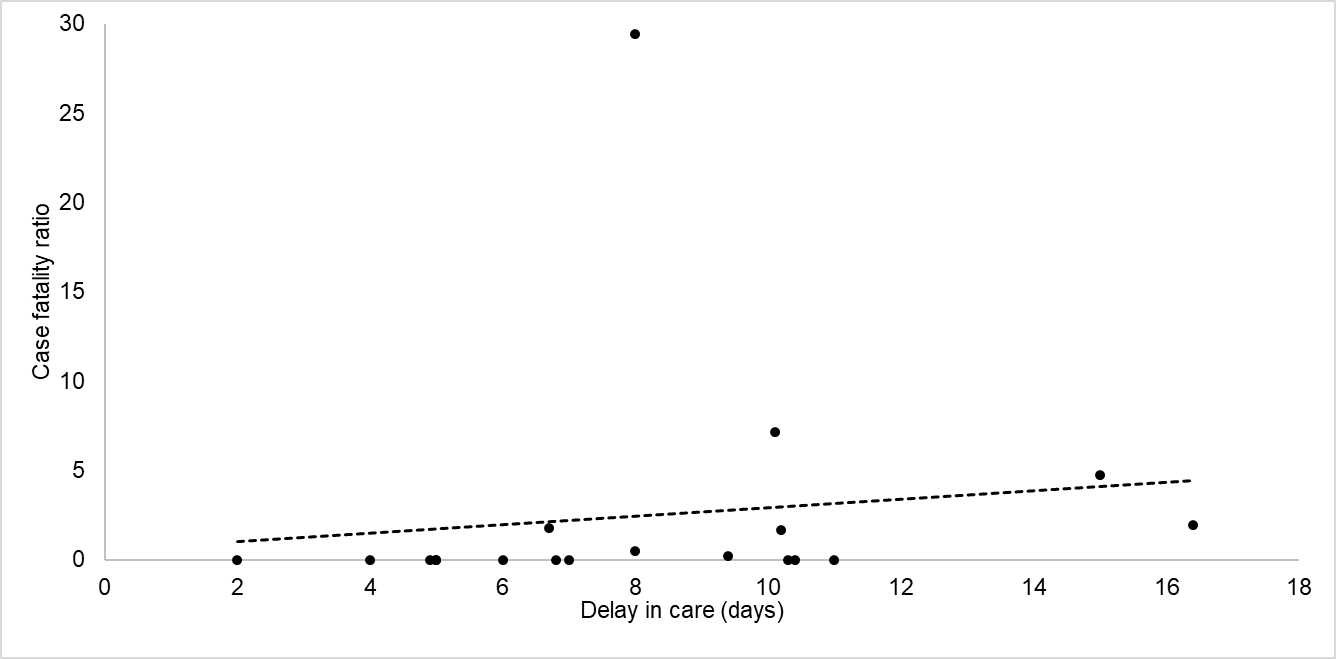
**

Trendline: y = 0.2352x + 0.5623

Pearson’s r = 0.1258 (p=0.6078)

**Figure S7. Scatterplot of case fatality ratio against delay in care with trendline, 18 eligible estimates from Asia and Africa regions removing a single outlier**

**
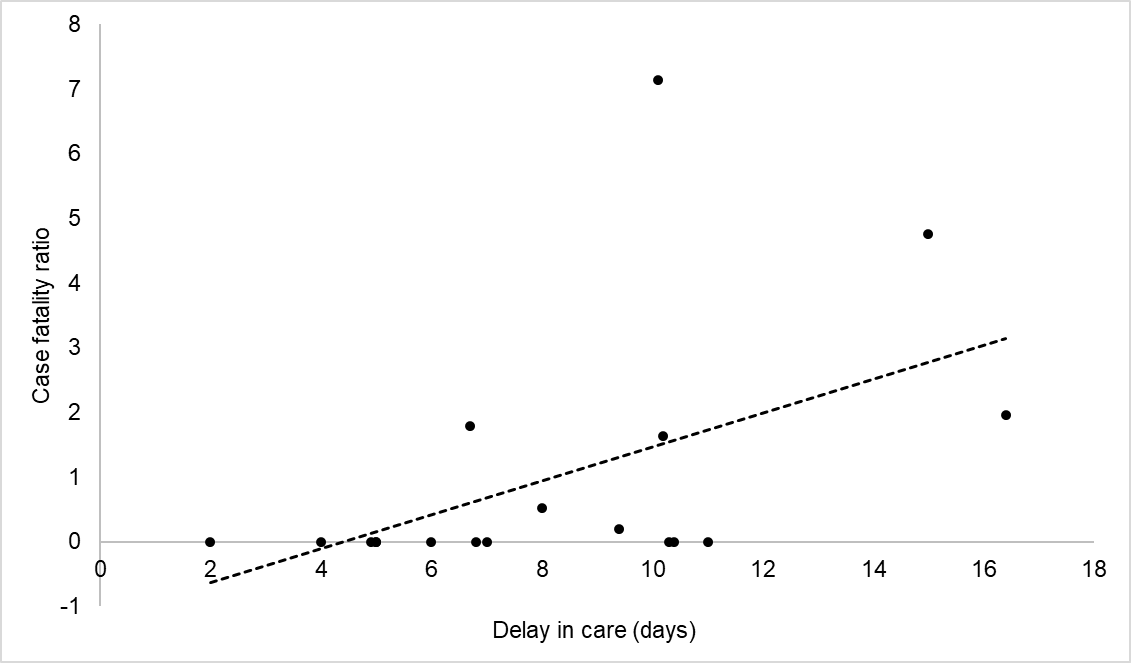
**

Trendline: y = 0.2617x - 1.154

Pearson’s r = 0.4976 (p=0.0356)

**Figure S8. Scatterplot of case fatality ratio against delay in care with trendline, 12 eligible estimates from the Asia region**

**
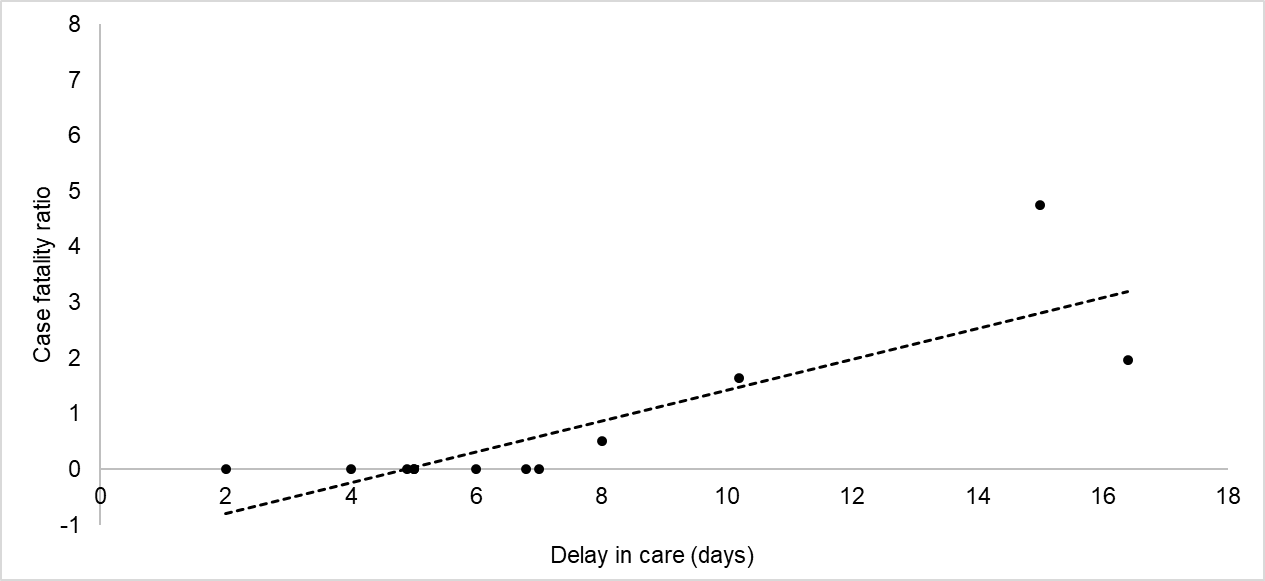
**

Trendline: y = 0.2774x - 1.3476

Pearson’s r = 0.8354 (p=0.0007)

**Figure S9. Scatterplot of case fatality ratio against delay in care with trendline, 7 eligible estimates from the Africa region**

**
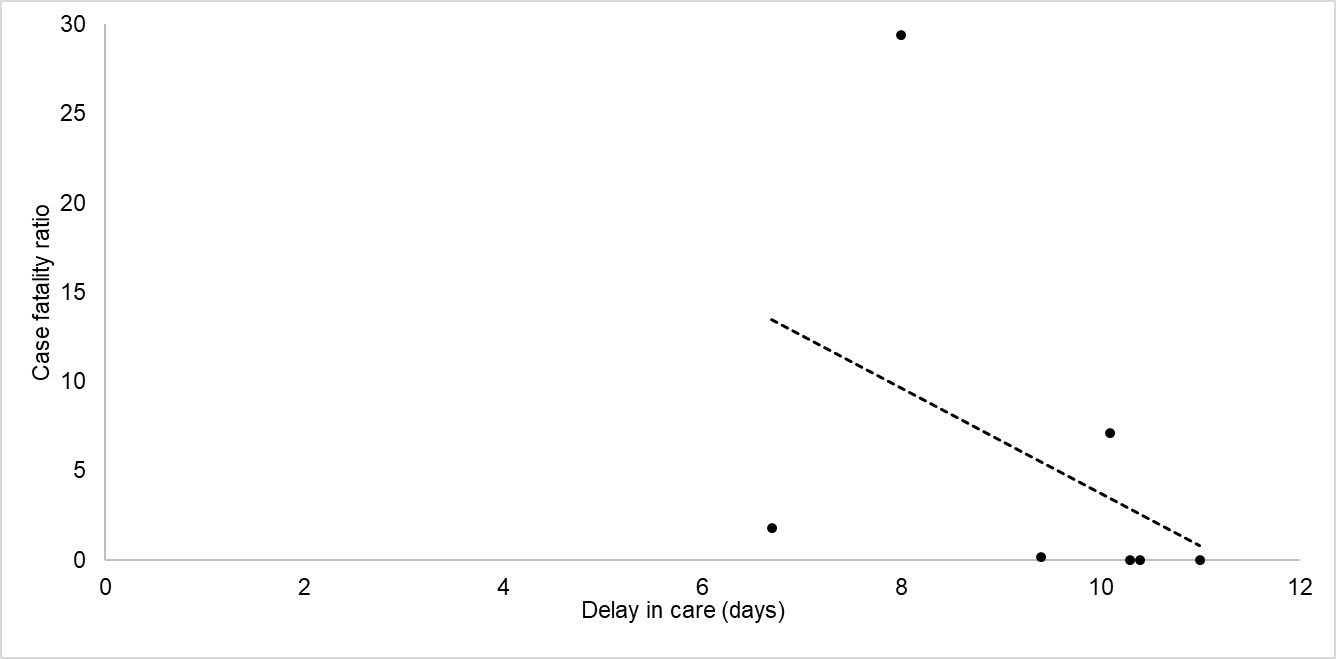
**

Trendline: y = -2.9449x + 33.23
Pearson’s r = -0.4160 (p=0.3533)

**Figure S10. Scatterplot of prevalence of complications against delay in care with trendline, 15 eligible estimates from the Asia and Africa regions**

**
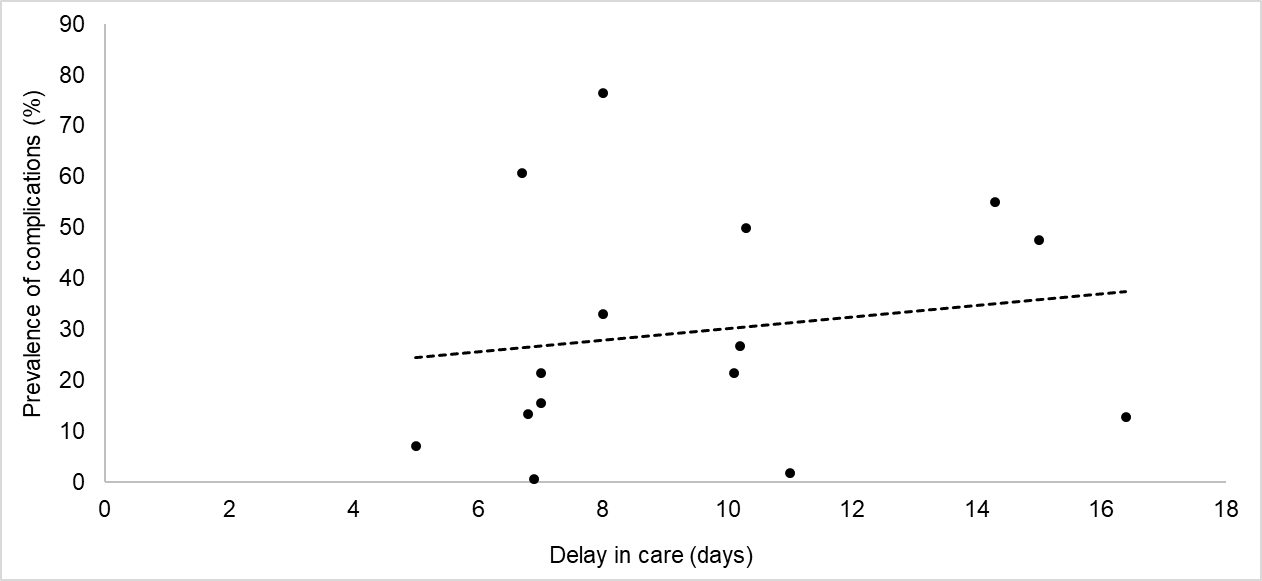
**

Trendline: y = 1.1383x + 18.727

Pearson’s r = 0.5968 (p=0.0685)

**References**

1. Abraham G, Teklu B. Typhoid fever: clinical analysis of 50 Ethiopian patients. *Ethiop Med J*. 1981;19(2):41-46.

2. Nesbitt A, Mirza NB. *Salmonella* septicaemias in Kenyan children. *J Trop Pediatr*. 1989;35(1):35-39. doi:10.1093/tropej/35.1.35

3. Muthumbi E, Morpeth SC, Ooko M, et al. Invasive salmonellosis in Kilifi, Kenya. *Clin Infect Dis Off Publ Infect Dis Soc Am*. 2015;61 Suppl 4:S290-301. doi:10.1093/cid/civ737

4. Breiman RF, Cosmas L, Njuguna H, et al. Population-based incidence of typhoid fever in an urban informal settlement and a rural area in Kenya: implications for typhoid vaccine use in Africa. *PloS One*. 2012;7(1):e29119. doi:10.1371/journal.pone.0029119

5. Gordon MA, Walsh AL, Chaponda M, et al. Bacteraemia and mortality among adult medical admissions in Malawi--predominance of non-Typhi salmonellae and Streptococcus pneumoniae. *J Infect*. 2001;42(1):44-49. doi:10.1053/jinf.2000.0779

6. Lepage P, Bogaerts J, Van Goethem C, et al. Community-acquired bacteraemia in African children. *Lancet Lond Engl*. 1987;1(8548):1458-1461. doi:10.1016/s0140-6736(87)92207-0

7. Ndayizeye L, Ngarambe C, Smart B, Riviello R, Majyambere JP, Rickard J. Peritonitis in Rwanda: Epidemiology and risk factors for morbidity and mortality. *Surgery*. 2016;160(6):1645-1656. doi:10.1016/j.surg.2016.08.036

8. Chalya PL, Mabula JB, Koy M, et al. Typhoid intestinal perforations at a University teaching hospital in Northwestern Tanzania: A surgical experience of 104 cases in a resource-limited setting. *World J Emerg Surg WJES*. 2012;7:4. doi:10.1186/1749-7922-7-4

9. Crump JA, Ramadhani HO, Morrissey AB, et al. Invasive bacterial and fungal infections among hospitalized HIV-infected and HIV-uninfected adults and adolescents in northern Tanzania. *Clin Infect Dis Off Publ Infect Dis Soc Am*. 2011;52(3):341-348. doi:10.1093/cid/ciq103

10. Crump JA, Ramadhani HO, Morrissey AB, et al. Invasive bacterial and fungal infections among hospitalized HIV-infected and HIV-uninfected children and infants in northern Tanzania. *Trop Med Int Health TM IH*. 2011;16(7):830-837. doi:10.1111/j.1365-3156.2011.02774.x

11. Mtove G, Amos B, von Seidlein L, et al. Invasive salmonellosis among children admitted to a rural Tanzanian hospital and a comparison with previous studies. *PloS One*. 2010;5(2):e9244. doi:10.1371/journal.pone.0009244

12. Conventi R, Pellis G, Arzu G, Nsubuga JB, Gelmini R. Intestinal perforation due to typhoid fever in Karamoja (Uganda). *Ann Ital Chir*. 2018;89:138-148.

13. Topley JM. Mild typhoid fever. *Arch Dis Child*. 1986;61(2):164-167. doi:10.1136/adc.61.2.164

14. Green SD, Cheesbrough JS. *Salmonella* bacteraemia among young children at a rural hospital in western Zaire. *Ann Trop Paediatr*. 1993;13(1):45-53. doi:10.1080/02724936.1993.11747624

15. Phoba M-F, De Boeck H, Ifeka BB, et al. Epidemic increase in *Salmonella* bloodstream infection in children, Bwamanda, the Democratic Republic of Congo. *Eur J Clin Microbiol Infect Dis Off Publ Eur Soc Clin Microbiol*. 2014;33(1):79-87. doi:10.1007/s10096-013-1931-8

16. Afifi S, Earhart K, Azab MA, et al. Hospital-based surveillance for acute febrile illness in Egypt: a focus on community-acquired bloodstream infections. *Am J Trop Med Hyg*. 2005;73(2):392-399.

17. Keenan JP, Hadley GP. The surgical management of typhoid perforation in children. *Br J Surg*. 1984;71(12):928-929. doi:10.1002/bjs.1800711203

18. Abdool Gaffar MS, Seedat YK, Coovadia YM, Khan Q. The white cell count in typhoid fever. *Trop Geogr Med*. 1992;44(1-2):23-27.

19. Khan M, Coovadia YM, Connolly C, Sturm AW. Influence of sex on clinical features, laboratory findings, and complications of typhoid fever. *Am J Trop Med Hyg*. 1999;61(1):41-46. doi:10.4269/ajtmh.1999.61.41

20. Keddy KH, Sooka A, Smith AM, et al. Typhoid fever in South Africa in an endemic HIV setting. *PloS One*. 2016;11(10):e0164939. doi:10.1371/journal.pone.0164939

21. Ouedraogo S, Ouangre E, Zida M. Epidemiological, clinical, and therapeutic features of typhoid intestinal perforation in a rural environment of Burkina Faso. *Med Sante Trop*. 2017;27(1):67-70. doi:10.1684/mst.2017.0661

22. Maltha J, Guiraud I, Kaboré B, et al. Frequency of severe malaria and invasive bacterial infections among children admitted to a rural hospital in Burkina Faso. *PloS One*. 2014;9(2):e89103. doi:10.1371/journal.pone.0089103

23. Guiraud I, Post A, Diallo SN, et al. Population-based incidence, seasonality and serotype distribution of invasive salmonellosis among children in Nanoro, rural Burkina Faso. *PloS One*. 2017;12(7):e0178577. doi:10.1371/journal.pone.0178577

24. Mock CN, Amaral J, Visser LE. Improvement in survival from typhoid ileal perforation. Results of 221 operative cases. *Ann Surg*. 1992;215(3):244-249. doi:10.1097/00000658-199203000-00008

25. van der Werf TS, Cameron FS. Typhoid perforations of the ileum. A review of 59 cases, seen at Agogo Hospital, Ghana, between 1982 and 1987. *Trop Geogr Med*. 1990;42(4):330-336.

26. Mock C, Visser L, Denno D, Maier R. Aggressive fluid resuscitation and broad spectrum antibiotics decrease mortality from typhoid ileal perforation. *Trop Doct*. 1995;25(3):115-117. doi:10.1177/004947559502500309

27. Abantanga FA. Complications of typhoid perforation of the ileum in children after surgery. *East Afr Med J*. 1997;74(12):800-802.

28. Oheneh-Yeboah M. Postoperative complications after surgery for typhoid ileal perforation in adults in Kumasi. *West Afr J Med*. 2007;26(1):32-36. doi:10.4314/wajm.v26i1.28300

29. Nilsson E, Olsson S, Regner S, et al. Surgical intervention for intestinal typhoid perforation. *Il G Chir*. 2019;40(2):105-111.

30. Laditan AA, Alausa KO. Problems in the clinical diagnosis of typhoid fever in children in the tropics. *Ann Trop Paediatr*. 1981;1(3):191-195. doi:10.1080/02724936.1981.11748087

31. Adesunkanmi AR, Ajao OG. The prognostic factors in typhoid ileal perforation: a prospective study of 50 patients. *J R Coll Surg Edinb*. 1997;42(6):395-399.

32. Tade AO, Ayoade BA, Olawoye AA. Pattern of presentation and management of typhoid intestinal perforation in Sagamu, South-West Nigeria: a 15 year study. *Niger J Med J Natl Assoc Resid Dr Niger*. 2008;17(4):387-390. doi:10.4314/njm.v17i4.37417

33. Osifo OD, Ogiemwonyi SO. Typhoid ileal perforation in children in Benin city. *Afr J Paediatr Surg AJPS*. 2010;7(2):96-100. doi:10.4103/0189-6725.62857

34. Ugwu BT, Yiltok SJ, Kidmas AT, Opaluwa AS. Typhoid intestinal perforation in north central Nigeria. *West Afr J Med*. 2005;24(1):1-6. doi:10.4314/wajm.v24i1.28152

35. Uba AF, Chirdan LB, Ituen AM, Mohammed AM. Typhoid intestinal perforation in children: a continuing scourge in a developing country. *Pediatr Surg Int*. 2007;23(1):33-39. doi:10.1007/s00383-006-1796-3

36. Edino ST, Mohammed AZ, Uba AF, et al. Typhoid enteric perforation in north western Nigeria. *Niger J Med J Natl Assoc Resid Dr Niger*. 2004;13(4):345-349.

37. Agu K, Nzegwu M, Obi E. Prevalence, morbidity, and mortality patterns of typhoid ileal perforation as seen at the University of Nigeria Teaching Hospital Enugu Nigeria: an 8-year review. *World J Surg*. 2014;38(10):2514-2518. doi:10.1007/s00268-014-2637-5

38. Nuhu A, Dahwa S, Hamza A. Operative management of typhoid ileal perforation in children. *Afr J Paediatr Surg AJPS*. 2010;7(1):9-13. doi:10.4103/0189-6725.59351

39. Talabi AO, Etonyeaku AC, Sowande OA, Olowookere SA, Adejuyigbe O. Predictors of mortality in children with typhoid ileal perforation in a Nigerian tertiary hospital. *Pediatr Surg Int*. 2014;30(11):1121-1127. doi:10.1007/s00383-014-3592-9

40. Usang UE, Inyang AW, Nwachukwku IE, Emehute J-DC. Typhoid perforation in children: an unrelenting plague in developing countries. *J Infect Dev Ctries*. 2017;11(10):747-752. doi:10.3855/jidc.9304

41. Ugochukwu AI, Amu OC, Nzegwu MA. Ileal perforation due to typhoid fever - review of operative management and outcome in an urban centre in Nigeria. *Int J Surg Lond Engl*. 2013;11(3):218-222. doi:10.1016/j.ijsu.2013.01.014

42. Obaro S, Lawson L, Essen U, et al. Community acquired bacteremia in young children from central Nigeria--a pilot study. *BMC Infect Dis*. 2011;11:137. doi:10.1186/1471-2334-11-137

43. Weeramanthri TS, Corrah PT, Mabey DC, Greenwood BM. Clinical experience with enteric fever in The Gambia, West Africa 1981-1986. *J Trop Med Hyg*. 1989;92(4):272-275.

44. Gbadoé AD, Lawson-Evi K, Dagnra AY, et al. [Pediatric salmonellosis at the Tokoin’s teaching hospital, Lomé (Togo)]. *Med Mal Infect*. 2008;38(1):8-11. doi:10.1016/j.medmal.2007.08.002

45. Ollé-Goig JE, Ruiz L. Typhoid fever in rural Haiti. *Bull Pan Am Health Organ*. 1993;27(4):382-388.

46. González Ojeda A, Pérez Ríos A, Rodríguez M, de la Garza Villaseñor L. [The surgical complications of typhoid fever: a report of 10 cases]. *Rev Gastroenterol Mex*. 1991;56(2):77-81.

47. Contreras R, Ferreccio C, Sotomayor V, et al. [Typhoid fever in school children: by what measures is the modification of the clinical course due to oral vaccination?]. *Rev Med Chil*. 1992;120(2):134-141.

48. Palacios Malmaceda PG, Vela Acosta JJ, Gutiérez Arrasco W. [Typhoid fever in children under 2 years of age]. *Bol Med Hosp Infant Mex*. 1981;38(3):473-483.

49. Chen YH, Chen TP, Tsai JJ, et al. Epidemiological study of human salmonellosis during 1991-1996 in southern Taiwan. *Kaohsiung J Med Sci*. 1999;15(3):127-136.

50. Ochiai RL, Acosta CJ, Danovaro-Holliday MC, et al. A study of typhoid fever in five Asian countries: disease burden and implications for controls. *Bull World Health Organ*. 2008;86(4):260-268. doi:10.2471/blt.06.039818

51. Chheng K, Carter MJ, Emary K, et al. A prospective study of the causes of febrile illness requiring hospitalization in children in Cambodia. *PloS One*. 2013;8(4):e60634. doi:10.1371/journal.pone.0060634

52. van den Bergh ET, Gasem MH, Keuter M, Dolmans MV. Outcome in three groups of patients with typhoid fever in Indonesia between 1948 and 1990. *Trop Med Int Health TM IH*. 1999;4(3):211-215. doi:10.1046/j.1365-3156.1999.43374.x

53. Punjabi NH, Taylor WRJ, Murphy GS, et al. Etiology of acute, non-malaria, febrile illnesses in Jayapura, northeastern Papua, Indonesia. *Am J Trop Med Hyg*. 2012;86(1):46-51. doi:10.4269/ajtmh.2012.10-0497

54. Chansamouth V, Thammasack S, Phetsouvanh R, et al. The aetiologies and impact of fever in pregnant inpatients in Vientiane, Laos. *PLoS Negl Trop Dis*. 2016;10(4):e0004577. doi:10.1371/journal.pntd.0004577

55. Choo KE, Razif A, Ariffin WA, Sepiah M, Gururaj A. Typhoid fever in hospitalized children in Kelantan, Malaysia. *Ann Trop Paediatr*. 1988;8(4):207-212. doi:10.1080/02724936.1988.11748572

56. Malik AS. Complications of bacteriologically confirmed typhoid fever in children. *J Trop Pediatr*. 2002;48(2):102-108. doi:10.1093/tropej/48.2.102

57. Abucejo PE, Capeding MR, Lupisan SP, et al. Blood culture confirmed typhoid fever in a provincial hospital in the Philippines. *Southeast Asian J Trop Med Public Health*. 2001;32(3):531-536.

58. Oh HM, Masayu Z, Chew SK. Typhoid fever in hospitalized children in Singapore. *J Infect*. 1997;34(3):237-242. doi:10.1016/s0163-4453(97)94283-3

59. Ahmad Hatib NA, Chong CY, Thoon KC, Tee NW, Krishnamoorthy SS, Tan NW. Enteric fever in a tertiary paediatric hospital: A retrospective six-year review. *Ann Acad Med Singapore*. 2016;45(7):297-302.

60. Thisyakorn U, Mansuwan P, Taylor DN. Typhoid and paratyphoid fever in 192 hospitalized children in Thailand. *Am J Dis Child 1960*. 1987;141(8):862-865. doi:10.1001/archpedi.1987.04460080048025

61. Wongsawat J, Pancharoen C, Thisyakorn U. Typhoid fever in children: experience in King Chulalongkorn Memorial Hospital. *J Med Assoc Thail Chotmaihet Thangphaet*. 2002;85(12):1247-1250.

62. Limpitikul W, Henpraserttae N, Saksawad R, Laoprasopwattana K. Typhoid outbreak in Songkhla, Thailand 2009-2011: clinical outcomes, susceptibility patterns, and reliability of serology tests. *PloS One*. 2014;9(11):e111768. doi:10.1371/journal.pone.0111768

63. Lin FY, Vo AH, Phan VB, et al. The epidemiology of typhoid fever in the Dong Thap Province, Mekong Delta region of Vietnam. *Am J Trop Med Hyg*. 2000;62(5):644-648. doi:10.4269/ajtmh.2000.62.644

64. Parry CM, Thompson C, Vinh H, et al. Risk factors for the development of severe typhoid fever in Vietnam. *BMC Infect Dis*. 2014;14:73. doi:10.1186/1471-2334-14-73

65. Butler T, Knight J, Nath SK, Speelman P, Roy SK, Azad MA. Typhoid fever complicated by intestinal perforation: a persisting fatal disease requiring surgical management. *Rev Infect Dis*. 1985;7(2):244-256. doi:10.1093/clinids/7.2.244

66. Brooks WA, Hossain A, Goswami D, et al. Bacteremic typhoid fever in children in an urban slum, Bangladesh. *Emerg Infect Dis*. 2005;11(2):326-329. doi:10.3201/eid1102.040422

67. Naheed A, Ram PK, Brooks WA, et al. Burden of typhoid and paratyphoid fever in a densely populated urban community, Dhaka, Bangladesh. *Int J Infect Dis IJID Off Publ Int Soc Infect Dis*. 2010;14 Suppl 3:e93-99. doi:10.1016/j.ijid.2009.11.023

68. Saha S, Islam MS, Sajib MSI, et al. Epidemiology of typhoid and paratyphoid: Implications for vaccine policy. *Clin Infect Dis Off Publ Infect Dis Soc Am*. 2019;68(Suppl 2):S117-S123. doi:10.1093/cid/ciy1124

69. Leung DT, Bogetz J, Itoh M, et al. Factors associated with encephalopathy in patients with *Salmonella* *enterica* serotype Typhi bacteremia presenting to a diarrheal hospital in Dhaka, Bangladesh. *Am J Trop Med Hyg*. 2012;86(4):698-702. doi:10.4269/ajtmh.2012.11-0750

70. Shahunja KM, Leung DT, Ahmed T, et al. Factors associated with non-typhoidal *Salmonella* bacteremia versus typhoidal *Salmonella* bacteremia in patients presenting for care in an urban diarrheal disease hospital in Bangladesh. *PLoS Negl Trop Dis*. 2015;9(9):e0004066. doi:10.1371/journal.pntd.0004066

71. Yu AT, Amin N, Rahman MW, Gurley ES, Rahman KM, Luby SP. Case-fatality ratio of blood culture-confirmed typhoid fever in Dhaka, Bangladesh. *J Infect Dis*. 2018;218(suppl_4):S222-S226. doi:10.1093/infdis/jiy543

72. Ali G, Rashid S, Kamli MA, Shah PA, Allaqaband GQ. Spectrum of neuropsychiatric complications in 791 cases of typhoid fever. *Trop Med Int Health TM IH*. 1997;2(4):314-318. doi:10.1111/j.1365-3156.1997.tb00145.x

73. Mukherjee P, Mukherjee S, Dalal BK, Haldar KK, Ghosh E, Pal TK. Some prospective observations on recent outbreak of typhoid fever in West Bengal. *J Assoc Physicians India*. 1991;39(6):445-448.

74. Chandra R, Srinivasan S, Nalini P, Rao RS. Multidrug resistant enteric fever. *J Trop Med Hyg*. 1992;95(4):284-287.

75. Mishra S, Patwari AK, Anand VK, et al. A clinical profile of multidrug resistant typhoid fever. *Indian Pediatr*. 1991;28(10):1171-1174.

76. Rao PS, Rajashekar V, Varghese GK, Shivananda PG. Emergence of multidrug-resistant *Salmonella* Typhi in rural southern India. *Am J Trop Med Hyg*. 1993;48(1):108-111. doi:10.4269/ajtmh.1993.48.108

77. Rasaily R, Dutta P, Saha MR, Mitra U, Lahiri M, Pal SC. Multi-drug resistant typhoid fever in hospitalised children. Clinical, bacteriological and epidemiological profiles. *Eur J Epidemiol*. 1994;10(1):41-46. doi:10.1007/BF01717450

78. Rodrigues C, Mehta A, Mehtar S, et al. Chloramphenicol resistance in *Salmonella* Typhi. Report from Bombay. *J Assoc Physicians India*. 1992;40(11):729-732.

79. Shetty AK, Mital SR, Bahrainwala AH, Khubchandani RP, Kumta NB. Typhoid hepatitis in children. *J Trop Pediatr*. 1999;45(5):287-290. doi:10.1093/tropej/45.5.287

80. Sinha A, Sazawal S, Kumar R, et al. Typhoid fever in children aged less than 5 years. *Lancet Lond Engl*. 1999;354(9180):734-737. doi:10.1016/S0140-6736(98)09001-1

81. Chaudhary P, Kumar R, Munjewar C, et al. Typhoid ileal perforation: a 13-year experience. *Healthc Low-Resour Settings*. 2015;3(1). doi:10.4081/hls.2015.4677

82. Kadhiravan T, Wig N, Kapil A, Kabra SK, Renuka K, Misra A. Clinical outcomes in typhoid fever: adverse impact of infection with nalidixic acid-resistant *Salmonella* Typhi. *BMC Infect Dis*. 2005;5:37. doi:10.1186/1471-2334-5-37

83. Sur D, von Seidlein L, Manna B, et al. The malaria and typhoid fever burden in the slums of Kolkata, India: data from a prospective community-based study. *Trans R Soc Trop Med Hyg*. 2006;100(8):725-733. doi:10.1016/j.trstmh.2005.10.019

84. Dheer G, Kundra S, Singh T. Clinical and laboratory profile of enteric fever in children in northern India. *Trop Doct*. 2012;42(3):154-156. doi:10.1258/td.2012.110442

85. Ganesh R, Janakiraman L, Vasanthi T, Sathiyasekeran M. Profile of typhoid fever in children from a tertiary care hospital in Chennai-South India. *Indian J Pediatr*. 2010;77(10):1089-1092. doi:10.1007/s12098-010-0196-9

86. Harichandran D, Dinesh KR. Antimicrobial susceptibility profile, treatment outcome and serotype distribution of clinical isolates of *Salmonella* *enterica* subspecies *enterica*: a 2-year study from Kerala, South India. *Infect Drug Resist*. 2017;10:97-101. doi:10.2147/IDR.S126209

87. Vala S, Shah U, Ahmad SA, Scolnik D, Glatstein M. Resistance patterns of typhoid Fever in children: A longitudinal community-based study. *Am J Ther*. 2016;23(5):e1151-1154. doi:10.1097/MJT.0000000000000094

88. Sur D, Barkume C, Mukhopadhyay B, Date K, Ganguly NK, Garrett D. A retrospective review of hospital-based data on enteric fever in India, 2014-2015. *J Infect Dis*. 2018;218(suppl_4):S206-S213. doi:10.1093/infdis/jiy502

89. Sucindar M, Kumaran SS. Profile of culture positive enteric fever in children admitted in a tertiary care hospital. *J Evol Med Dent Sci*. 2017;6(88):6112-6117.

90. Kurlberg G, Frisk B. Factors reducing mortality in typhoid ileal perforation. *Trans R Soc Trop Med Hyg*. 1991;85(6):793-795. doi:10.1016/0035-9203(91)90458-b

91. Mathura KC, Gurubacharya DL, Shrestha A, Pant S, Basnet P, Karki DB. Clinical profile of typhoid patients. *Kathmandu Univ Med J KUMJ*. 2003;1(2):135-137.

92. Petersiel N, Shresta S, Tamrakar R, et al. The epidemiology of typhoid fever in the Dhulikhel area, Nepal: A prospective cohort study. *PloS One*. 2018;13(9):e0204479. doi:10.1371/journal.pone.0204479

93. Bhutta ZA. Impact of age and drug resistance on mortality in typhoid fever. *Arch Dis Child*. 1996;75(3):214-217. doi:10.1136/adc.75.3.214

94. Sulaiman K, Sarwari AR. Culture-confirmed typhoid fever and pregnancy. *Int J Infect Dis IJID Off Publ Int Soc Infect Dis*. 2007;11(4):337-341. doi:10.1016/j.ijid.2006.09.007

95. Khan MI, Soofi SB, Ochiai RL, et al. Epidemiology, clinical presentation, and patterns of drug resistance of *Salmonella* Typhi in Karachi, Pakistan. *J Infect Dev Ctries*. 2012;6(10):704-714. doi:10.3855/jidc.1967

96. Owais A, Sultana S, Zaman U, Rizvi A, Zaidi AKM. Incidence of typhoid bacteremia in infants and young children in southern coastal Pakistan. *Pediatr Infect Dis J*. 2010;29(11):1035-1039.

97. Qamar FN, Yousafzai MT, Sultana S, et al. A retrospective study of laboratory-based enteric fever surveillance, Pakistan, 2012-2014. *J Infect Dis*. 2018;218(suppl_4):S201-S205. doi:10.1093/infdis/jiy205

98. Saeed N, Usman M, Khan EA. An overview of extensively drug-resistant *Salmonella* Typhi from a tertiary care hospital in Pakistan. *Cureus*. 2019;11(9):e5663. doi:10.7759/cureus.5663

99. Magsi AM, Iqbal M, Khan S, Parveen S, Khan MI, Shamim M. Frequency, presentation and outcomes of typhoid ileal perforation. *Indo Am J Pharm Sci*. 2019;6(6):1-5.

100. Aziz S, Malik L. Emergence of multi-resistant enteric infection in a paediatric unit of Karachi, Pakistan. *JPMA J Pak Med Assoc*. 2018;68(12):1848-1850.

101. Dworkin J, Saeed R, Mykhan H, et al. Burden of typhoid fever in Sulaimania, Iraqi Kurdistan. *Int J Infect Dis IJID Off Publ Int Soc Infect Dis*. 2014;27:70-73. doi:10.1016/j.ijid.2014.07.005

102. Carmeli Y, Raz R, Schapiro JM, Alkan M. Typhoid fever in Ethiopian immigrants to Israel and native-born Israelis: a comparative study. *Clin Infect Dis Off Publ Infect Dis Soc Am*. 1993;16(2):213-215. doi:10.1093/clind/16.2.213

103. Atamanalp SS, Aydinli B, Ozturk G, Oren D, Basoglu M, Yildirgan MI. Typhoid intestinal perforations: twenty-six year experience. *World J Surg*. 2007;31(9):1883-1888. doi:10.1007/s00268-007-9141-0

104. Seçmeer G, Kanra G, Cemeroğlu AP, Ozen H, Ceyhan M, Ecevit Z. *Salmonella* Typhi infections. A 10-year retrospective study. *Turk J Pediatr*. 1995;37(4):339-341.

105. Gedik E, Girgin S, Taçyildiz IH, Akgün Y. Risk factors affecting morbidity in typhoid enteric perforation. *Langenbecks Arch Surg*. 2008;393(6):973-977. doi:10.1007/s00423-007-0244-8

106. Hosoglu S, Aldemir M, Akalin S, Geyik MF, Tacyildiz IH, Loeb M. Risk factors for enteric perforation in patients with typhoid Fever. *Am J Epidemiol*. 2004;160(1):46-50. doi:10.1093/aje/kwh172

107. Prieto López I, de la Fuente Aguado J, González Díaz I, et al. [Infection by *Salmonella* Typhi in the southern area of Ponteverde]. *An Med Interna Madr Spain 1984*. 1994;11(2):71-73.

108. S AG, Parry CM, Crump JA, et al. A retrospective study of patients with blood culture-confirmed typhoid fever in Fiji during 2014-2015: epidemiology, clinical features, treatment and outcome. *Trans R Soc Trop Med Hyg*. 2019;113(12):764-770. doi:10.1093/trstmh/trz075

109. Rogerson SJ, Spooner VJ, Smith TA, Richens J. Hydrocortisone in chloramphenicol-treated severe typhoid fever in Papua New Guinea. *Trans R Soc Trop Med Hyg*. 1991;85(1):113-116. doi:10.1016/0035-9203(91)90180-7
